# Extracellular vimentin mimics VEGF and is a target for anti-angiogenic immunotherapy

**DOI:** 10.1038/s41467-022-30063-7

**Published:** 2022-05-23

**Authors:** Judy R. van Beijnum, Elisabeth J. M. Huijbers, Karlijn van Loon, Athanasios Blanas, Parvin Akbari, Arno Roos, Tse J. Wong, Stepan S. Denisov, Tilman M. Hackeng, Connie R. Jimenez, Patrycja Nowak-Sliwinska, Arjan W. Griffioen

**Affiliations:** 1 Amsterdam UMC location Vrije Universiteit Amsterdam, Angiogenesis Laboratory, Department of Medical Oncology, Amsterdam, The Netherlands; 2 Cancer Center Amsterdam, Cancer Biology and Immunonology, Amsterdam, The Netherlands; 3 CimCure BV, The Hague, The Netherlands; 4 Veterinary Referral Centre Korte Akkeren, Gouda, The Netherlands; 5 Department of Biochemistry, Cardiovascular Research Institute Maastricht (CARIM), University of Maastricht, Maastricht, The Netherlands; 6 Amsterdam UMC location Vrije Universiteit Amsterdam, Oncoproteomics Laboratory, Department of Medical Oncology, Amsterdam, The Netherlands; 7 School of Pharmaceutical Sciences, Faculty of Science, University of Geneva, Geneva, Switzerland; 8 Institute of Pharmaceutical Sciences of Western Switzerland, University of Geneva, Geneva, Switzerland; 9 Translational Research Center in Oncohaematology, University of Geneva, Geneva, Switzerland

**Keywords:** Cancer immunotherapy, Tumour angiogenesis, Tumour immunology

## Abstract

Anti-angiogenic cancer therapies possess immune-stimulatory properties by counteracting pro-angiogenic molecular mechanisms. We report that tumor endothelial cells ubiquitously overexpress and secrete the intermediate filament protein vimentin through type III unconventional secretion mechanisms. Extracellular vimentin is pro-angiogenic and functionally mimics VEGF action, while concomitantly acting as inhibitor of leukocyte-endothelial interactions. Antibody targeting of extracellular vimentin shows inhibition of angiogenesis in vitro and in vivo. Effective and safe inhibition of angiogenesis and tumor growth in several preclinical and clinical studies is demonstrated using a vaccination strategy against extracellular vimentin. Targeting vimentin induces a pro-inflammatory condition in the tumor, exemplified by induction of the endothelial adhesion molecule ICAM1, suppression of PD-L1, and altered immune cell profiles. Our findings show that extracellular vimentin contributes to immune suppression and functions as a vascular immune checkpoint molecule. Targeting of extracellular vimentin presents therefore an anti-angiogenic immunotherapy strategy against cancer.

## Abstract (web summary)

The pro-tumorigenic effects of vimentin have been attributed to intracellular functions in tumour cells so far. Here, the authors show that tumour endothelial cells can secrete vimentin as a pro-angiogenic factor and that targeting of vimentin can be used as an immunotherapeutic strategy.

## Introduction

The tumor microenvironment (TME) is highly immunosuppressive, which is heavily mediated by the aberrant tumor vasculature^1,2^. As a consequence of continuous exposure to tumor-derived growth factors, tumor endothelial cells (ECs) become anergic to inflammatory cytokines, resulting in a non-adhesive vasculature and subsequent evasion from immunity^3–5^.

The current commercial success of targeting the vasculature indirectly—through interference with tumor-derived angiogenic growth factors by antibodies and tyrosine kinase inhibitors—is overshadowed by the occurrence of drug-induced resistance, resulting from the adaptation and alternative growth factor production of tumor cells^6,7^. We have shown that direct targeting of tumor endothelium, by vaccination or antibodies towards tumor endothelial-specific markers, is a highly effective strategy for inhibiting tumor growth and can potentially overcome EC anergy^8–11^. As such, targeting tumor blood vessels has the capacity to improve immunotherapy and may even act as immunotherapy in itself^5,12^.

The intermediate filament protein vimentin is elaborately investigated and known for its intracellular structural properties and contribution to enhanced malignancy of tumors by its involvement in epithelial to mesenchymal transition (EMT) and metastasis^13^. In recent years, extracellular roles for vimentin have been proposed^8,14,15^ and in this study, we demonstrate that ECs externalize vimentin, in an effort to promote angiogenesis and, at the same time, escape from immunity. The latter involves a role as a vascular immune checkpoint, shielding the vasculature from leukocyte interactions. Importantly, both passive and active antibody-based immunotherapies against extracellular vimentin are shown to specifically and safely inhibit tumor vascularization and tumor growth. This is demonstrated in several preclinical models, as well as in a clinical study in client-owned domestic dogs presenting with spontaneous bladder carcinoma. The anti-vimentin approach overcomes tumor immune suppression by enhancing infiltration, and altering the composition, of immune cells in the tumor area. This effect is mediated by regulation of ICAM1 expression and endothelial adhesiveness, as well as through mimicking VEGF actions including enhancing VEGFR signaling. Our data show that extracellular vimentin is a vascular immune checkpoint molecule and that targeting this bioavailable marker provides a double-edged sword in cancer therapy, simultaneously alleviating immune suppression and repressing tumor angiogenesis.

## Results

### Tumor ECs overexpress and secrete vimentin, a universal marker of the tumor vasculature

**Fig. 1:**
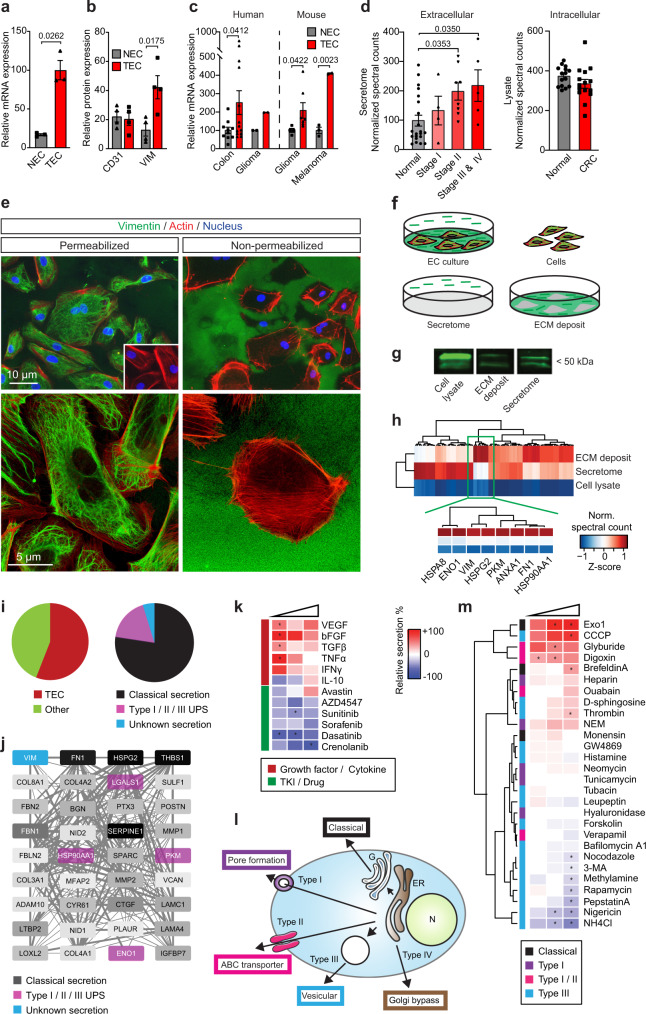
Vimentin is overexpressed in tumor endothelial cells and is present extracellularly. **a**, **b** Vimentin mRNA (**a**; *n* = 3; qPCR) and protein (**b**; *n* = 4; flow cytometry) expression in isolated endothelial cells (EC) from human colon tumor (TEC) and normal colon (NEC). **c** Vimentin mRNA expression in isolated EC from human (colon, *n* = 13; glioma, *n* = 2) and murine (glioma, *n* = 7; melanoma, *n* = 2) tumors. **d** Proteomics analysis of human normal colon and colorectal cancer tissues for extracellular vimentin in secretome (left panel; *n* = 21 (normal), *n* = 4 (Stage I), *n* = 8 (Stage II), *n* = 5 (Stage III & IV)) and total intracellular vimentin (right panel; *n* = 15 (normal), *n* = 15 (CRC Stage I–IV)). Data are presented as mean ± SEM in **a**–**d**. *p* values represent paired *t* test (**a**, **c**, **d** right panel), unpaired *t* test (**b**), and one-way ANOVA (**d** left panel). **e** Immunofluorescent staining of fixated and permeabilized HUVEC (left panels) and live intact HUVEC (right panels). Inset: negative control. Representative images of at least three independent experiments are shown. **f** Schematic representation of vimentin localization (in green). **g** Western blotting of total cell lysate, ECM deposit, and secretome of HUVEC. Representative sections of at least three independent experiments are shown. **h** Global proteomics analysis (*n* = 1) of HUVEC lysate, secretome, and ECM deposit. **i** (Left) Proportion of known tumor EC markers (TEC, red) among externalized proteins. (Right) Secretion mechanisms among externalized proteins. **j** Protein–protein interaction analysis using STRING of externalized TEC markers. Opacity levels of the nodes are proportional to secretion abundance. **k** Effect of angiogenesis inhibitors and cytokines on vimentin secretion. Relative secretion is color-coded according to the legend right of the panel, and agent types are color-coded according to the legend below the panel. **l** Schematic of different cellular protein secretion pathways. **m** Effect of different protein secretion mediators on vimentin secretion. Legend as in **k**. Data are color-coded as mean values of relative secretion in **k** and **m**; numbers of samples are presented in the Source Data file. *p < 0.05 based on Kruskal–Wallis test with Dunn’s multiple comparison test correction for **k** and **m**. Source data are provided as a Source Data file.

Vimentin was found to be overexpressed in the endothelium of a wide array of human tumor types and in syngeneic and xenograft animal tumors, by transcript and protein analysis (Fig. 1a–c, Supplementary Fig. 1a–c). In colorectal tumor tissues, vimentin protein is abundantly present in the vessel wall, although other mesenchymal cell types such as resident immune cells also express the protein (Supplementary Fig. 1b). Vimentin gene expression was found to be strongly positively correlated with focal adhesion and extracellular matrix (ECM) turnover, hallmark processes in the tumor microenvironment during tumor angiogenesis, as well as with other described tumor endothelial markers, e.g., galectin-1 (Supplementary Fig. 1e)^8,11,16^. Vimentin expression in ECs was inducible by exposure to angiogenic factors, while expression was reduced in the presence of angiogenesis inhibitors (Supplementary Fig. 1d). It was also found to be causally related to activation of ECs, as silencing of vimentin by siRNA (Supplementary Fig. 1f), dose-dependently resulted in angiogenesis inhibition in vitro, predominantly evidenced by reduced migration and sprouting capacity (Supplementary Fig. 1g–j).

While fixed and permeabilized ECs show the characteristic abundant filamentous network of vimentin, also staining of depositions surrounding the cells was observed, which was better visible in non-permeabilized cells and after non-enzymatic removal of the cells (Fig. 1e, Supplementary Fig. 2a–d). The presence of vimentin in cell lysate, matrix depositions, and conditioned medium (secretome; Fig. 1f) was investigated by western blot analysis. This demonstrated that all samples contained the 54 kDa full-length vimentin and showed the characteristic multiple band pattern that is due to posttranslational modifications and/or cellular proteolytic enzyme activity (Fig. 1g, Supplementary Fig. 2e)^17,18^. For the different cells used in this study, intracellular vimentin was quantified by flow cytometry, and extracellular vimentin was quantified in the secretome by ELISA. Intracellular vimentin expression varied between the cells (Supplementary Fig. 2j), while secreted vimentin was detected within this panel exclusively in the secretome of ECs (Supplementary Fig. 2k). Indeed, it was previously shown that vimentin is not readily secreted from colorectal tumor cell lines^19^. However, we observed cancer stage-related presence of extracellular vimentin in the secretome of human colorectal tumors, while total, intracellular vimentin levels did not differ between the normal colon and colorectal cancer (Fig. 1d). These observations substantiate the significance of vimentin secretion in malignancies.

### Vimentin is secreted through non-classical pathways

The above results were further confirmed using proteomics analysis of HUVEC lysate, secretome, and ECM deposit (Fig. 1h, Supplementary Fig. 2f). Vimentin was among the most abundantly externalized proteins from HUVEC, along with fibronectin 1 (FN1). Coverage of tryptic peptides over the length of the total protein sequence was comparable among all sample types, which confirms the presence of full-length secreted vimentin (Supplementary Fig. 2g). Interestingly, the majority of the externalized proteins have previously been recognized as markers of tumor ECs by us and others (Fig. 1i, j)^8,16,20^. Furthermore, ~25% of the externalized proteins belonged to the class of non-classically secreted proteins, essentially lacking the sequence features that are ascribed to classically (Golgi and ER-mediated) secreted proteins (Fig. 1i, Supplementary Fig. 2h, i)^21^. Moreover, the most abundantly secreted proteins (present in the ECM deposit, secretome, or both), are highly interconnected as demonstrated by protein-protein interaction analysis (Fig. 1j). This may indicate that common, hitherto unknown secretion mechanisms play a role in the externalization of these proteins from the cell.

We observed that stimulation of ECs with angiogenic growth factors increased vimentin secretion, whereas anti-angiogenic agents tended to decrease its secretion (Fig. 1k), suggesting that vimentin secretion is associated with the activation state of ECs. Moreover, blockade of classical secretion mechanisms through inhibition of ER and Golgi by brefeldin A and monensin did not inhibit vimentin secretion (Fig. 1m), as was also observed for secretion of IL-1β^22^.

To further unravel the endothelial vimentin secretion mechanism, we screened for the effects of 28 known regulators of various cellular secretion mechanisms (Fig. 1l, m; Supplementary Table 1), at concentrations that did not affect cell viability. Interestingly, inhibitors of type III unconventional protein secretion (UPS) pathways strongly inhibited vimentin secretion, suggesting the involvement of secretory organelles. Notably, interference with lysosomal and autophagy functions (e.g., NH4Cl, nigericin, pepstatin A) inhibited vimentin secretion, while disruptors of classical secretion (e.g., Exo1, brefeldin A) and of membrane potential or direct membrane transport (e.g., CCCP, digoxin, glyburide) tended to stimulate secretion. Such features are known for other unconventionally secreted proteins^22–24^.

### Extracellular vimentin promotes a pro-angiogenic phenotype

**Fig. 2:**
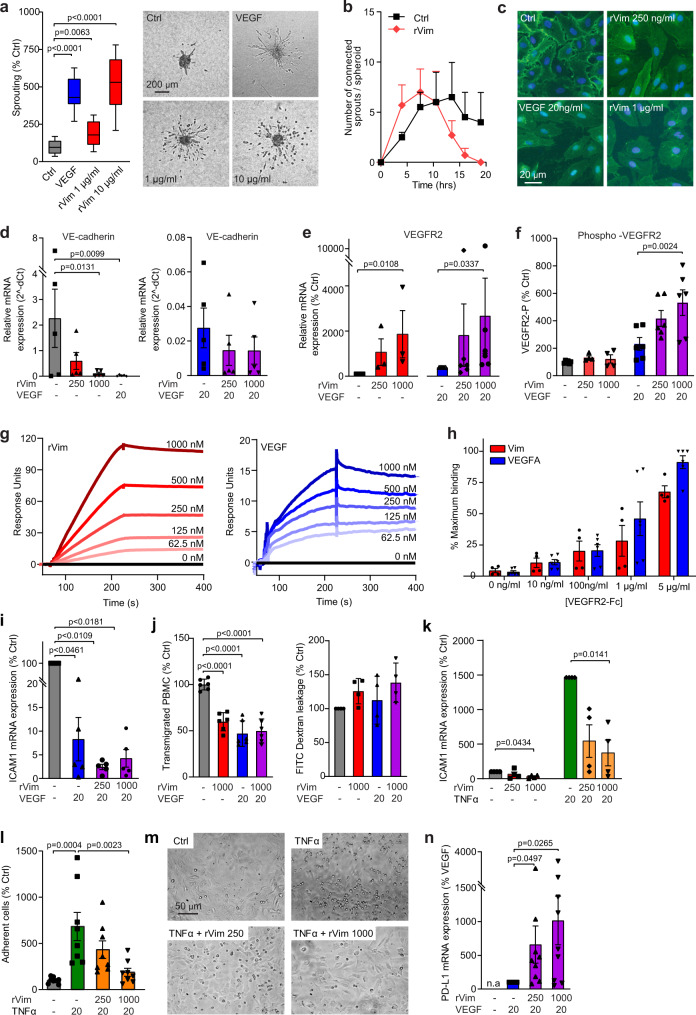
Extracellular vimentin promotes an anti-adhesive and pro-migratory endothelial phenotype. **a**, **b** Sprouting from collagen embedded HUVEC spheroids in the presence of recombinant vimentin (rVim), after 16 h (**a**; *n* = 4 independent experiments) and in time (**b**; *n* = 3). Box plots (**a**) represent medians ± 10–90th percentiles. XY-plot (**b**) represents mean + SEM. *p* values represent one-way ANOVA with Bonferroni correction for multiple comparisons. **c** Immunofluorescence for VE-cadherin expression in HUVEC after treatment with VEGF and rVim. VE-cadherin expression is depicted in green, nuclei are stained in blue with DAPI. Representative images of at least three independent experiments are shown. **d**, **e** VE-cadherin (**d**) and VEGFR2 (**e**) mRNA expression in HMEC-1. *n* = 5 (**d**), *n* = 3 (**e**) independent experiments. **f** VEGFR2 phosphorylation measurement in HMEC-1 by ELISA. *n* = 3 independent experiments. Bar graphs in **d**–**f** represent means ± SEM. *p* values represent one-way ANOVA with Bonferroni correction for multiple comparisons (**d**, **f**) or Kruskal–Wallis test with Dunn’s correction for multiple comparisons (**e**). **g** Surface plasmon resonance analysis of binding or rVim (left panel) and VEGF (right panel) to coated VEGFR2-Fc. *n* = 1. **h** Detection of binding of VEGFR2-Fc to coated rVim (*n* = 4) or VEGF (*n* = 6) using ELISA. Bar graphs represent means ± SEM. **i** ICAM1 mRNA expression in HMEC-1 after treatment with rVim in the presence of VEGF. *n* = 5 independent experiments. Bar graphs represent means ± SEM. *p* values represent Kruskal–Wallis test with Dunn’s correction for multiple comparisons. **j** Transmigration of PBMC over a HUVEC monolayer in a transwell assay (left panel) in the presence of rVim and/or VEGF. *n* = 3 independent experiments. *p* values represent one-way ANOVA with Bonferroni correction for multiple comparisons. Leakage of FITC-dextran (right panel) over a HUVEC monolayer. *n* = 4 independent experiments. *p* values represent Kruskal–Wallis test with Dunn’s correction for multiple comparisons. Bar graphs represent means ± SEM. **k** ICAM1 mRNA expression in HMEC-1 after treatment with rVim and/or TNFα. *n* = 4 independent experiments. Bar graphs represent means ± SEM. *p* values represent Kruskal–Wallis test with Dunn’s correction for multiple comparisons. **l**, **m** Adhesion of Jurkat T cells to TNFα stimulated HUVEC in the presence or absence of rVim; representative images (**m**) and quantification (**l**; *n* = 4 different donors). *p* values represent one-way ANOVA with Bonferroni correction for multiple comparisons. Bar graphs represent means ± SEM. **n** PD-L1 mRNA expression in HMEC-1 after treatment with rVim and/or VEGF (*n* = 4 independent experiments). Bar graphs represent means ± SEM, *p* values represent Kruskal–Wallis test with Dunn’s correction for multiple comparisons. All rVim concentrations are in ng/ml unless otherwise indicated. VEGF and TNFα were used at 20 ng/ml. Representative images are shown in **c** and **m**. Source data are provided as a Source Data file.

Exposure of ECs to recombinant extracellular vimentin dose-dependently increased sprouting of ECs in collagen gels (Fig. 2a), while cell viability and migration were not significantly affected (Supplementary Fig. 3a, b). Notably, we observed that in the presence of extracellular vimentin, though not in response to VEGF, invaded cells lost connectivity and migrated into the collagen gel individually, rather than as connected tubes (Fig. 2a). Using time-lapse imaging of this assay system, and quantification of invading tubes vs. invading individual cells, we noted that tubes do form in the presence of extracellular vimentin, but disassemble over time (Fig. 2b). Similarly, in the presence of extracellular vimentin cells tended to migrate more as individual cells into a scratched area in a monolayer (Supplementary Fig. 3b). In line with these observations, when ECs were plated onto Matrigel, normally resulting in honeycomb-like structures (meshes), we observed inhibition of this alignment in the presence of vimentin. This phenotype was only apparent, however, when cells were seeded immediately in the presence of vimentin, while the addition of vimentin after primary adhesion and alignment of the cells after 2 hours had no effect (Supplementary Fig. 3c). Importantly, these apparent anti-adhesive effects of recombinant vimentin were partially counteracted by the addition of anti-vimentin antibodies (Supplementary Fig. 3d, e). Taken together, these observations show that extracellular vimentin impairs cell-cell and cell-matrix interactions.

When monolayers of ECs were treated with vimentin, intercellular gaps were observed. This was accompanied by a redistribution of the major cell-cell adhesion molecule VE-cadherin, away from the cell surface and towards a more cytoplasmic localization, similar to that observed after treatment of ECs with VEGF (Fig. 2c)^25^. Moreover, vimentin and VEGF significantly inhibited VE-cadherin mRNA expression. The combination of VEGF and vimentin further suppressed VE-cadherin expression, although this did not reach statistical significance (Fig. 2d). When we evaluated whether recombinant vimentin induced VEGF expression in EC to account for these effects, we observed that somewhat counterintuitively, both VEGF and vimentin suppress VEGF mRNA expression (Supplementary Fig. 3f). These parallel effects suggest that vimentin functionally mimics VEGF. We, therefore, suspected that vimentin might modulate VEGF receptor expression and/or function. Indeed, treatment of EC with VEGF alone or in combination with vimentin stimulated VEGFR2 mRNA expression (Fig. 2e). Importantly, vimentin, in combination with VEGF, increased VEGFR2 phosphorylation (Fig. 2f), though this did not affect the presence of VEGFR2 on the cell surface (Supplementary Fig. 3g). This suggests that extracellular vimentin directly binds to VEGFR2. To support this hypothesis, we carried out SPR biosensor analysis, by which we show that vimentin binds immobilized VEGFR2 in a dose-dependent manner (Fig. 2g). Additionally, this analysis was confirmed by binding of VEGFR2 to immobilized vimentin and VEGF in ELISA (Fig. 2h) and reciprocal spot blot analyses (Supplementary Fig. 3h). Together, these data provide evidence for the involvement of vimentin in regulating the cell-cell adhesive properties of the vasculature through modulation of VEGF-VEGFR signaling. Sharing of VEGF and vimentin effects by signaling through VEGFRs is further addressed in the next paragraph.

### Extracellular vimentin inhibits vascular immune functions

We demonstrated in the past that angiogenic growth factors, like VEGF, are potent suppressors of endothelial adhesion molecules, such as ICAM1 and VCAM1^26^. Indeed, VEGF was shown to potently suppress ICAM1 expression, which is even more pronounced after additional exposure to extracellular vimentin (Fig. 2i). In addition, transmigration of human PBMCs over a HUVEC monolayer in a transwell system was inhibited in the presence of extracellular vimentin, VEGF, and the combination thereof (Fig. 2j). These effects were not due to direct effects on the viability of PBMCs, nor a consequence of generally enhanced permeability (Fig. 2j, Supplementary Fig. 3i, j). Independently, extracellular vimentin also clearly suppressed endothelial ICAM1 expression, which was partially prevented in the presence of TNFα (Fig. 2k, Supplementary Fig. 3k). We could exclude this to be mediated by direct blockade of TNFα receptors, as even in the absence of TNFα this suppression was observed. Functionally, it resulted in impaired TNFα induced adhesion of T cells to endothelial monolayers (Fig. 2l, m).

Whereas endothelial ICAM1 and VCAM1 expression are pivotal for effective immune responses, in contrast, endothelial expression of checkpoint molecules such as PD-L1 (CD274) can hamper immune responses. PD-L1 can interact with PD-1 on effector T cells and thereby inactivate those, resulting in immune evasion^27,28^. While PD-L1 was not detected in unstimulated ECs, exposure to VEGF resulted in a detectable expression. Moreover, additional exposure to extracellular vimentin significantly enhanced the expression of PD-L1 on ECs (Fig. 2n). These data further corroborate our observations that extracellular vimentin can potentiate VEGF-VEGFR signaling and functionally mimic VEGF actions.

### Anti-vimentin antibodies inhibit angiogenesis and tumor growth

**Fig. 3:**
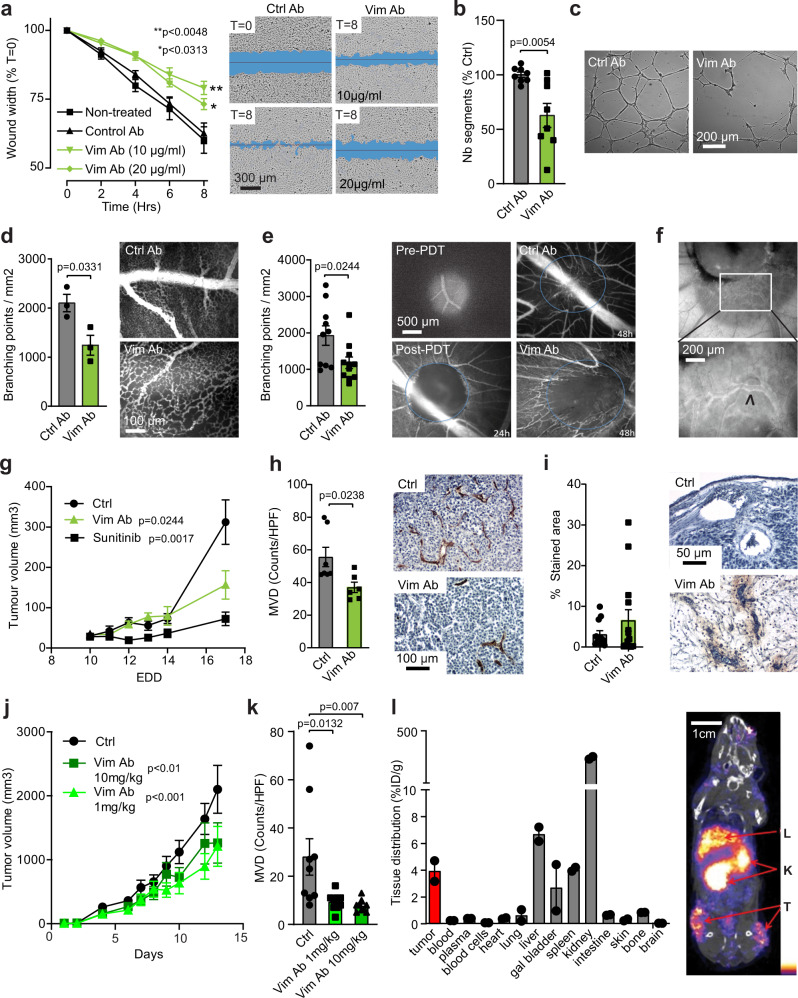
Anti-vimentin antibodies inhibit angiogenesis. **a** HUVEC scratch wound analysis in the presence of anti-vimentin antibodies (Vim Ab). *n* = 4 different donors. Data represent means ± SEM. *p* values represent two-way ANOVA with Dunnett’s correction for multiple comparisons for treatment. Representative images are shown in the right panel. **b**, **c** Tube formation of HUVEC on Matrigel in the presence of anti-vimentin antibodies (Vim Ab) or control antibodies (Ctrl Ab) *n* = 4 different donors. Bar graphs represent means ± SEM. *p* values represent unpaired *t* test. Representative images are shown. **d**, **e** Vessel density in physiological CAMs (**d**) and after photodynamic therapy (PDT) (**e**), treated with Vim Ab or Ctrl Ab. *n* = 3 (**d**), and *n* = 10 (Ctrl Ab) *n* = 11 (Vim Ab) (**e**) eggs/group. Bar graphs represent means ± SEM. *p* values represent unpaired *t* test. Representative images are shown to the right of the graphs. **f** Fluorescently labeled Vim Ab after i.v. injection localizes to the tumor vasculature in the CAM spheroid (arrow). Bottom panel: magnification of white box. Representative images of a single experiment are shown. **g**–**i** HCT116 xenograft tumor growth on the CAM, topically treated daily with 100 µl antibody or 2 µM sunitinib. **g** Tumor growth. *n* = 8 (Vim Ab), *n* = 9 (Ctrl, sunitinib) eggs/group. Data represent means ± SEM. *p* values represent two-way ANOVA with Dunnett’s correction for multiple comparisons for treatment. **h** Microvessel density (MVD) in Vim Ab (*n* = 7) and Ctrl (*n* = 6) treated tumors on the CAM. Data represent means ± SEM. *p* values unpaired *t* test. **i** Detection of tumor-homed antibodies in *n* = 12 (Ctrl Ab) and *n* = 14 (Vim Ab) images/group. Representative images are shown. **j** Passive Vim Ab therapy of B16F10 melanoma tumor growth in mice. *n* = 10 mice/group, *p* values represent two-way ANOVA. **k** MVD in *n* = 3 fields/tumor for *n* = 3 mice/group. Data represent means ± SEM. *p* values represent one-way ANOVA with Bonferroni correction. **l** Tissue distribution of 89-Zr labeled anti-vimentin nanobodies in mice (*n* = 2) with B16F10 melanoma (T = tumor, K = kidney, L = liver). Data represent means ± SEM. Source data are provided as a Source Data file.

Antagonizing secreted vimentin using anti-vimentin antibodies resulted in dose-dependent inhibition of EC scratch wound migration, sprouting into collagen, and mesh formation on Matrigel, but not EC viability (Fig. 3a–c; Supplementary Fig. 3l–n). In accordance, while in vivo angiogenesis in the chicken chorioallantoic membrane (CAM) was induced by the application of recombinant vimentin (Supplementary Fig. 3o), suppression of angiogenesis was observed in the presence of anti-vimentin antibodies that are reactive with chicken vimentin, in both naïve models and after angiogenesis induction by photodynamic therapy (Fig. 3d, e; Supplementary Fig. 3p, q)^29^. Furthermore, intravital imaging of FITC-labeled anti-vimentin antibodies injected in tumor-grafted CAMs showed localization of the antibodies to the tumor vessel wall (Fig. 3f). Treatment of xenografted human CRC on the CAM with anti-vimentin antibodies inhibited both tumor growth and vascular density in the tumors (Fig. 3g, h), and resulted in increased necrosis (Supplementary Fig. 4a). Moreover, these antibodies could be detected in the perivasculature in excised tumor sections, confirming effective homing to the tumor vasculature (Fig. 3i).

In a mouse model of subcutaneously grafted B16F10 melanoma, anti-vimentin antibodies inhibited tumor growth and tumor vessel density (Fig. 3j, k). A more detailed analysis of the tumor tissues shows that following anti-vimentin antibody treatment of the mice, tumor vascular integrity is impaired, resulting in the less pronounced demarcation of blood vessels and dispersion of erythrocytes into the tumor parenchyma (Supplementary Fig. 4b). Furthermore, vascular Icam1 expression is increased (Supplementary Fig. 4c), and analysis of infiltrating T cells and macrophages by immunostaining for Cd3 and F4/80, respectively, suggest a minor increase in immune infiltrate after treatment, although this did not reach statistical significance (Supplementary Fig. 4d). In addition, myeloid cells, stained for Cd11b, appeared to remain confined to the tumor periphery in untreated mice, whereas upon anti-vimentin antibody treatment Cd11b cells could be observed in the tumor core as well (Supplementary Fig. 4e).

Finally, a clear accumulation of a Zirconium-89 labeled anti-vimentin nanobody in immunoPET imaging was observed in tumors (Fig. 3l), showing the promise of monitoring ongoing tumor angiogenesis with anti-vimentin antibodies, and confirming the selective extracellular bioavailability of vimentin in tumor vasculature. Importantly, as shown in the HCT116 CAM and B16F10 mouse tumor models presented in this section, as well as in the models described below, effective targeting of tumor vascular vimentin is independent of the intracellular expression level of vimentin in the tumor cells (Supplementary Fig. 2j) as vimentin is dominantly expressed in the vasculature in vivo and detected in the tumor secretome (Supplementary Fig. 5f, g).

Taken together, these antibody-based studies show the potential of inhibiting tumor angiogenesis and tumor growth by targeting extracellular vimentin secreted by the tumor endothelium, which we approach by vaccination as presented below.

### Active immunization against extracellular vimentin inhibits tumor growth

**Fig. 4:**
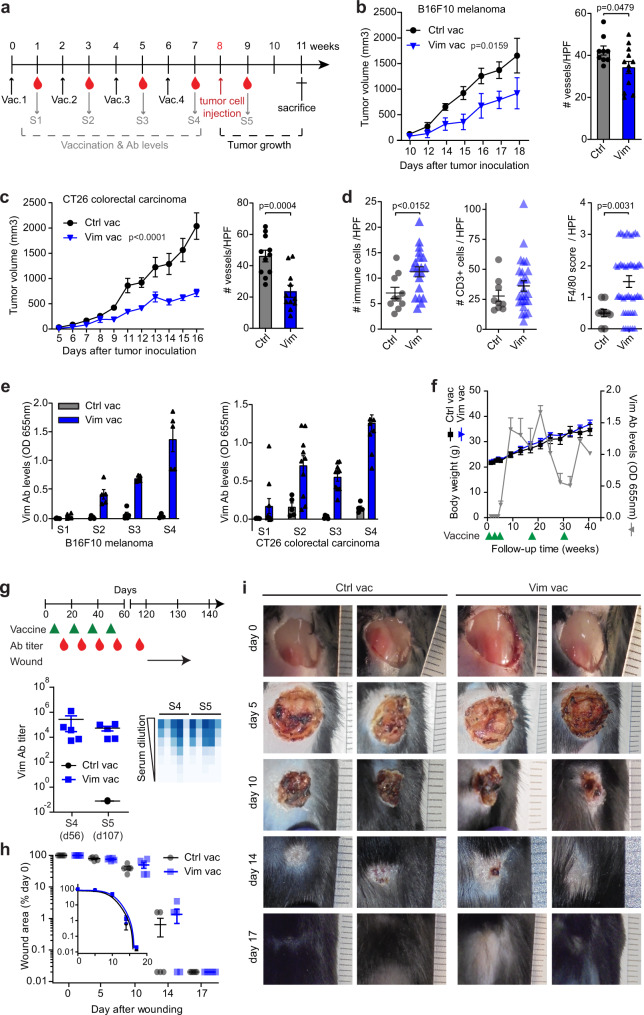
Vaccination against vimentin inhibits tumor growth. **a** Vaccination scheme. **b** B16F10 tumor growth in vaccinated C57BL/6 mice (left panel, *n* = 5 mice/group) and microvessel density (MVD, right panel; *n* = 3 fields/tumor for *n* = 3 (Ctrl Vac) and *n* = 4 (Vim Vac) mice/group). Data represent means ± SEM. *p* values represent two-way ANOVA with Dunnett’s correction for multiple comparisons for treatment (left panel) and unpaired *t* test (right panel). **c** CT26 tumor growth in vaccinated (BALB/c) mice (left panel, *n* = 5 mice (Ctrl Vac) and *n* = 10 mice (Vim Vac)) and MVD (right panel, *n* = 3 fields/tumor for *n* = 2 (Ctrl Vac) and *n* = 4 (Vim Vac) mice/group). Data represent means ± SEM. *p* values represent two-way ANOVA with Dunnett’s correction for multiple comparisons for treatment (left panel) and unpaired *t* test (right panel) **d** Quantifications of immune cell infiltration into CT26 tumor tissue. H&E stain, left panel, *n* = 5 fields/tumor for *n* = 2 (Ctrl Vac) and *n* = 4 (Vim Vac) mice/group, ×400 magnification; Cd3+ cells, middle panel and F4/80- score, right panel, *n* = 3 fields/tumor for *n* = 3 (Ctrl Vac) and *n* = 9 (Vim Vac) mice/group, ×200 magnification. Data represent means ± SEM. *p* values represent unpaired *t* test (H&E, Cd3) and Mann–Whitney *U* test (F4/80). **e** Vimentin antibody levels following vaccination. B16F10: *n* = 5 mice/group; CT26: *n* = 5 (Ctrl Vac) and n = 10 (Vim Vac) mice/group. Data represent means ± SEM. **f** Long-term evaluation of vaccinated mice. *n* = 5 mice/group. Data represent means ± SEM. **g**–**i** Skin wound healing in vaccinated mice. Vaccination scheme and antibody titers (data represent means ± SEM), with a heatmap representation of ELISA signals after serial dilution of the individual sera (**g**). Wound closure over time (**h**, data represent means ± SEM) with representative images shown (**i**). *n* = 5 mice/group. Source data are provided as a Source Data file.

We have previously described the development of a vaccination strategy (iBoost technology) to evoke a humoral immune response to self-antigens, based on immunization with the self-antigen conjugated to an engineered bacterial protein^9^. Here, we chose this technology to target vimentin by vaccination as a strategy against cancer (Fig. 4a, Supplementary Fig. 5a). A primary vaccination and three booster vaccinations with a potent immune adjuvant were given at 2-week intervals. In two different syngeneic preclinical models, i.e. B16F10 melanoma grafted s.c. in C57BL/6 and CT26 colorectal carcinoma grafted s.c. in BALB/c, tumor growth was significantly reduced (Fig. 4b, c; left panels). All animals in both models developed an adequate anti-vimentin antibody response over time and showed no signs of adverse effects based on monitoring of body weight, histopathology, or behavioral determinants (Fig. 4e, Supplementary Fig. 5b, c). Further analysis of excised tumors showed reduced vascular density in the vimentin vaccination group as compared to the control group (Fig. 4b, c; right panels), while the amount of infiltrating immune cells, notably macrophages, was increased (Fig. 4d), confirming effectiveness through inhibition of angiogenesis and stimulation of antitumor immunity.

To further establish the safety of the vaccination strategy, mice were kept hyperimmune for 40 weeks. Antibody levels were determined every 4 weeks, and mice were revaccinated when these dropped on two consecutive time points. Vimentin-vaccinated mice responded well to revaccination by increasing antibody levels, and body weight development did not differ from that of control vaccinated mice (Fig. 4f). No behavioral differences were observed and post-mortem histopathological analysis of major organs revealed no morphological differences between the different vaccination groups (Supplementary Fig. 5d). In addition, wound healing studies in mice were performed, to exclude therapy-related complications in this process. Full-thickness 8-mm puncture wounds were made in the skin of immunized and control mice, and wound healing was monitored over time. Wounds in all mice recovered over a period of 17 days and no differences in wound closure were observed between mice vaccinated with vimentin and control vaccinated mice (Fig. 4g–i, Supplementary Fig. 5e). Together, these data show that targeting extracellular vimentin through active immunization is safe and effective.

### Antagonizing extracellular vimentin overcomes immune suppression

**Fig. 5:**
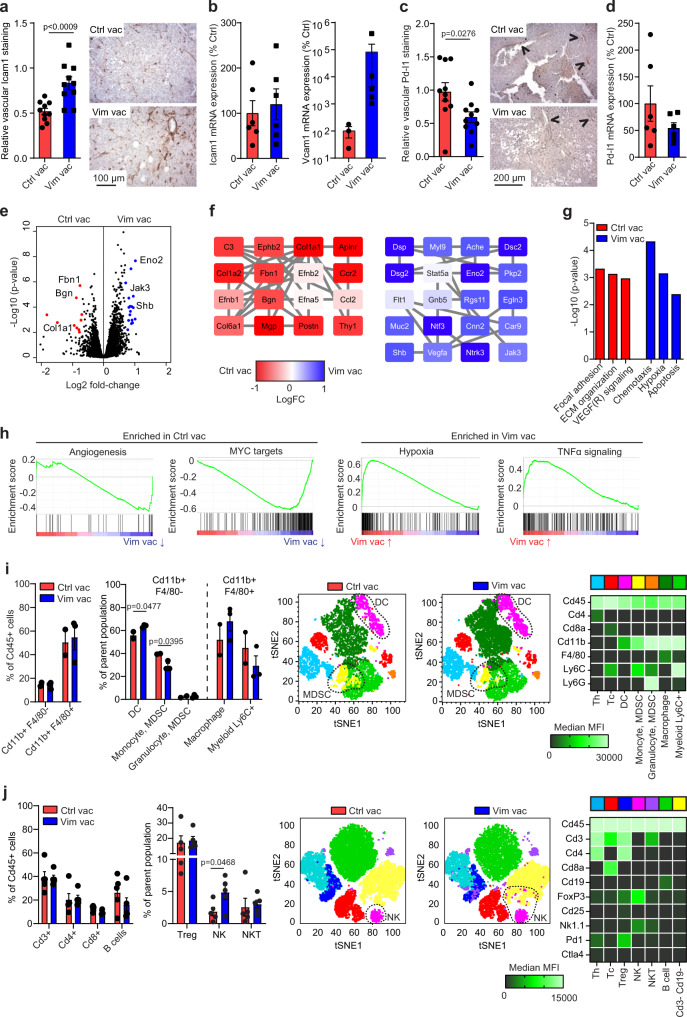
Vimentin functions as a vascular immune checkpoint. **a** Vascular Icam1 protein in B16F10 tumors (*n* = 10 tumors/group) of vaccinated mice. Data represent means ± SEM. *p* values represent unpaired *t* test. **b** Icam1 and Vcam1 mRNA expression in B16F10 tumors (*n* = 3 tumors/group, two independent analyses) of vaccinated mice. Data represent means ± SEM. **c** Vascular Pd-l1 protein expression in B16F10 tumors of vaccinated mice. *n* = 10 tumors/group. Data represent means ± SEM. *p* values represent unpaired *t* test. Arrows indicate Pd-l1-positive blood vessels. **d** Pd-l1 mRNA expression in B16F10 tumors of vaccinated mice. *n* = 3 tumors/group, two independent analyses. Data represent means ± SEM. **e** Volcano plot of RNAseq analysis of control and vimentin-vaccinated mice. **f** Protein-protein interaction analysis of differentially expressed genes. **g** Enriched gene ontologies of differentially expressed genes. **h** Gene set enrichment analysis (GSEA) on RNAseq data. Enriched gene sets in control (left panels) or vimentin (right panels) vaccinated mice. **i**, **j** Profiling of immune cell subsets in B16F10 tumors of control and vimentin-vaccinated mice by flow cytometry. Antibody panels for the detection of myeloid (**i**, *n* = 2, Ctrl Vac and *n* = 3 Vim Vac) and lymphoid (**j**, *n* = 5, Ctrl Vac and *n* = 5 Vim Vac) cells were applied and used to classify different cellular subsets (left panels; bar graphs represent means ± SEM. *p* values represent unpaired *t* test). Heatmaps (right panels) show the median fluorescence intensity of the subsets and populations are similarly color-coded in the tSNE plots (middle panels). Source data are provided as a Source Data file.

As shown above, impaired endothelial-leukocyte interactions, mediated by extracellular vimentin, appear to be overcome by therapeutic targeting of vimentin. To further unravel the relevance of these findings, we evaluated the expression of Icam1 in tumors (B16F10) of vimentin-vaccinated mice. Immunohistochemical staining revealed a clear induction of vascular Icam1 expression following vaccination against vimentin (Fig. 5a), in line with the effects of passive antibody therapy (Supplementary Fig. 4c). While the total Icam1 mRNA expression showed only a minor increase, probably due to Icam1 expression in non-ECs (Fig. 5b), mRNA expression of the blood vessel-specific adhesion molecule Vcam1 was markedly increased in tumors of vimentin-vaccinated mice (Fig. 5b). Concordantly, staining of B16F10 tumor sections of vimentin-vaccinated mice for Pd-l1 revealed that vascular expression was reduced (Fig. 5c), as was supported by mRNA analysis (Fig. 5d). Together, these data illustrate that antagonizing extracellular vimentin promotes a more immune permissive tumor vasculature.

Global gene expression analysis of control vs. vimentin-vaccinated B16F10 mouse tumors (Fig. 5e–g) revealed that hypoxia, as well chemokine signaling signatures (including IL-2, IL-7, IL-9, and TNFα), were induced after vimentin vaccination, supporting an immune-stimulatory role for anti-vimentin vaccination. These data are corroborated by profiling of soluble cytokines in the secretomes of B16F10 tumors from vaccinated mice, which point to a global subtle increase in pro-inflammatory cytokine expression (e.g., IL-1b, IL-6, MCP-1) and a decrease in immunosuppressive IL-10 following vaccination against vimentin (Supplementary Fig. 6a). In contrast, angiogenesis and oncogenic signaling (including Myc, E2F, and Pten) were dominant in control tumors (Fig. 5h), in which we also observed dominant expression of known tumor endothelial markers, e.g., Bgn, Col1a1 (Fig. 5e, f)^8,16^. In silico deconvolution analysis of bulk RNAseq data using mMCP-counter analysis^30^, which provides estimates of cellular phenotypes within a gene expression data set, further showed that tumors of vimentin-vaccinated mice showed an enhanced presence of immune cell subsets, and a decrease in the presence of stromal components, most notably vasculature (Supplementary Fig. 6b). This global analysis underscores a reversal of tumor phenotype in vimentin-vaccinated mice.

Tumor vaccination is a form of active immunotherapy that mobilizes both the innate and the adaptive arms of the immune system^31^. To elucidate how vaccination against extracellular vimentin impacts innate antitumor immunity, we first assessed the differences in the frequency of intratumoral myeloid subsets among vimentin-immunized and control vaccinated mice. Interestingly, vimentin vaccination induced higher rates of dendritic cells (DC) and reduced the frequency of monocytic myeloid-derived suppressor cells (M-MDSC) within tumors (Fig. 5i). The frequency of granulocytic myeloid-derived suppressor cells (G-MDSC) was comparable between the two groups, although we noticed a shift from Cd11b^+^F4/80^+^Ly6C^+^ myeloid cells towards macrophages (Cd11b^+^F4/80^+^Ly6C^−^) in the vaccination group compared to the control group (Fig. 5i). The observed changes in the myeloid compartment (DC, M-MDSC, macrophages) prompted us to further examine potential alterations in the lymphoid subsets upon vaccination, since lymphoid cells are indicative of the adaptive antitumor immunity. Although vimentin vaccination did not seem to significantly amend the percentage of most infiltrated T and B cells, consistent with our immunohistochemistry-based observations, we identified a marked increase of intratumoral natural killer (NK) cells in vimentin-vaccinated mice relative to control vaccinated mice (Fig. 5j). In addition, we noted slightly decreased Pd-1 expression on intratumoral NKT cells following vimentin vaccination (Supplementary Fig. 6c).

Finally, we aimed to establish the relationship between the expression of vimentin and immune infiltrate in clinically relevant settings. Large-scale gene expression data sets of human CRC, glioma, and melanoma were selected for in silico profiling of the immune cell landscape by digital flow cytometry using CIBERSORT^32^. In all data sets, a clear reduction in T-cell subsets is seen in high vimentin expressing tumors, whereas the relative presence of macrophages, most notably the pro-angiogenic M2 type, is increased in tumors with high vimentin expression levels (Supplementary Fig. 6d).

Altogether, these data indicate that extracellular vimentin directly contributes to an immunosuppressive tumor microenvironment and provides the rationale for the effective induction of immune infiltrate in tumors of vimentin-vaccinated mice.

### Vimentin vaccination is clinically effective and safe

To demonstrate the therapeutic potential of extracellular vimentin vaccination, an efficacy study was performed in client-owned dogs with spontaneous transitional cell carcinoma (TCC) of the bladder. TCC in domestic dogs is highly aggressive and is relatively insensitive to chemo- and radiation therapy, with reported historic 50% survival varying between 181 and 244 days^33–36^.

**Fig. 6:**
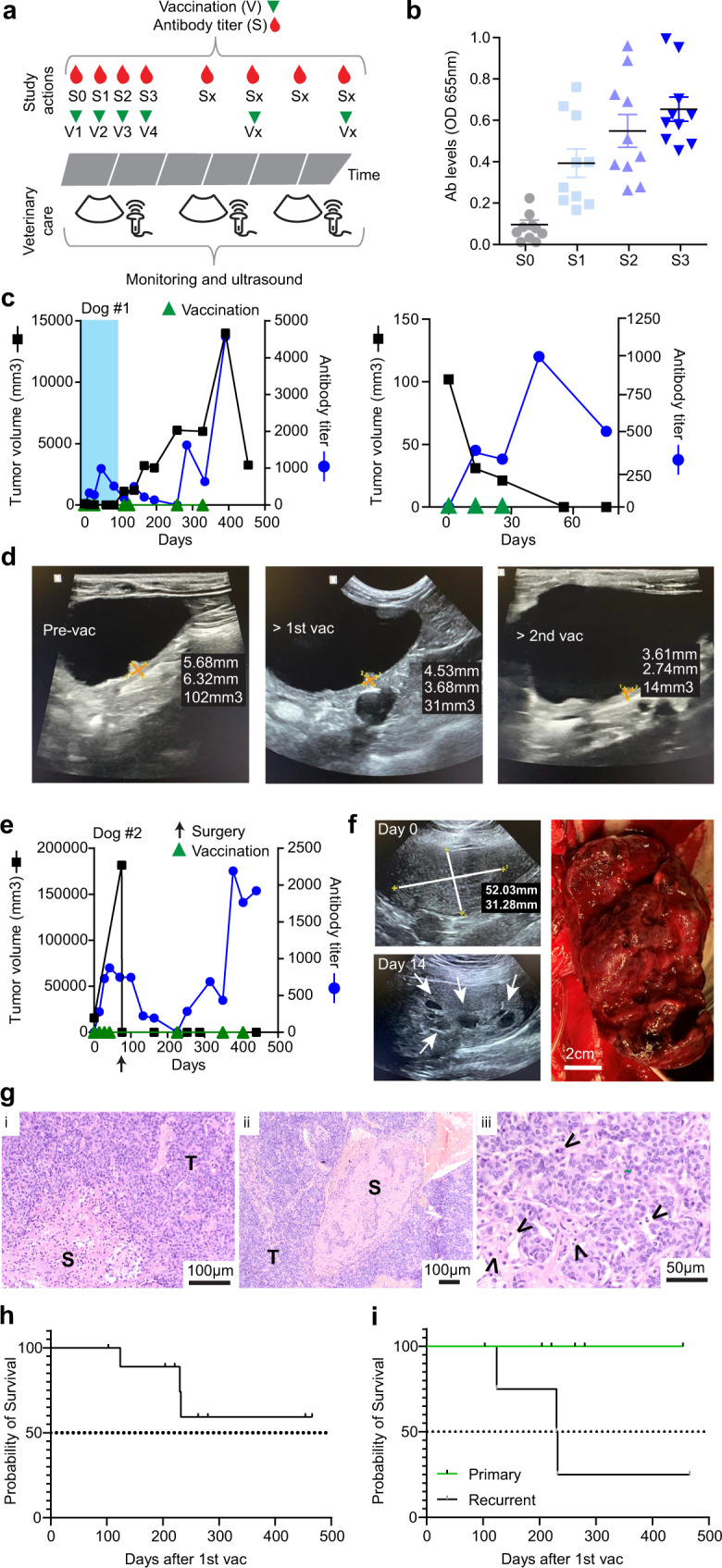
Vimentin vaccination is effective in dog patients. **a** Study setup. **b** Anti-vimentin antibody level development of the 10 dogs. Data represent means ± SEM. **c**, **d** Case report (tumor volume and antibody titers) of dog #1. The shaded area in the left panel is expanded in the right graph in (**c**). Ultrasound images during the first month of treatment (**d**). **e**–**g** Case report (tumor volume and antibody titers) of dog #2. Black arrow: surgical debulking (**e**). Ultrasound images of the tumor at the start of the vaccinations (top left) and prior to surgery (bottom left). White arrows point to necrotic areas. Right panel, the macroscopic image of the excised tumor (**f**). H&E staining of the excised tumor. S and T in (i, ii) indicate stroma and tumor, respectively. Arrows in (iii) point to the presence of immune cell infiltrate (**g**). **h**, **i** Survival analysis of all dogs (**h**, *n* = 10) or split out (**i**) for primary TCC (green; *n* = 6) and recurrent TCC (black; *n* = 4) in vimentin-vaccinated dogs. Source data are provided as a Source Data file.

Ten dogs are included in this interim analysis (Table 1; Supplementary Table 2), of which 4 presented with recurrent disease. Dogs were vaccinated as described, and continued to receive meloxicam. After 3-4 vaccinations with 2-week intervals (Fig. 6a), all dogs developed adequate anti-vimentin antibody levels and all dogs experienced a clinical response to the therapy, with an established best response of stable disease in 7/10 dogs and complete or partial remission in 3/10 dogs (Fig. 6b; Table 1; Supplementary Fig. 7a, b)^37^. The first dog (#1) presented with a small tumor (~100 mm^3^) of recurrent TCC. Two weeks after the first vaccination, when circulating antibodies were clearly present, the tumor showed regression (Fig. 6c, d). After three vaccinations no residual tumor mass was detected anymore (Fig. 6c, right panel). Revaccination after relapse resulted again in stasis and subsequent regression. In another dog (#2, Fig. 6e–g), multiple necrotic areas within the tumor tissue were observed already after the first vaccination (Fig. 6f). This necessitated the removal of the primary tumor. Surgical wounds healed normally and the tumor did not grow back until this writing (>350 days following surgery, Fig. 6e). H&E staining revealed the clear presence of immune infiltrate in both stromal and tumor areas (Fig. 6g).

**Table 1:** Dog patient characteristics.

Parameter	Specification	
Age (yr)	10.6 (mean)	7.0–14.2 (range)
Weight (kg)	23.1 (mean)	4.4–68 (range)
Sex	Male (castrated)	4
	Female (spayed)	5
	Female (intact)	1
Primary/recurrent	Primary	6
	Recurrent	4
Location	Fundus	4
	Apex	2
	Other	4
Pretreatment	Surgery	2
	PDT	3
	None	5
Best response	CR/PR	3
	SD	7

Other than minor injection site reactivity and short episodes of mild fever (<2 days, maximum AE grade 2 in 2/10 dogs) after the vaccinations, there were no major signs of adverse effects and all dogs tolerated the treatment well^38^. During the course of the study, one dog treated for recurrent TCC was euthanized due to progressive disease and one dog with recurrent TCC was euthanized post-surgery (Supplementary Table 2). One dog was euthanized for non-TCC-related causes, and one was withdrawn from the study, as per owner's decision. Survival analysis of the dogs included in this interim analysis shows improvement over historical survival, especially in dogs with primary disease (Fig. 6h, i). Taken together, this clinical pilot study demonstrated the efficacy and safety of the application of vaccination against vimentin in a clinical setting in large mammals, and will guide the development of clinical application in human patients.

## Discussion

This study unveils a pivotal role for vimentin in the biology of cancer. By excretion of this cytoskeletal protein by tumor ECs, tumor angiogenesis is facilitated and an escape mechanism from immunity is provided. We report that vimentin is externalized by non-classical secretion pathways from activated tumor ECs, where it is deposited in the tumor cell-vasculature interface and used by ECs to support of migration and formation of new vasculature. Intriguingly, extracellular vimentin seems to phenocopy the effects of VEGF. Moreover, we show that extracellular vimentin contributes to an immunosuppressive tumor environment by suppressing leukocyte adhesion molecules such as ICAM1 and inducing immune checkpoint molecules on the endothelium, thereby impairing effective leukocyte infiltration and potentially contributing to immune exhaustion. Finally, we demonstrate that by both passive (monoclonal antibodies) and active (vaccination) immunotherapy tumor growth is inhibited and antitumor immunity is augmented. This study demonstrates the feasibility and efficacy, as well as the safety, of targeting vimentin as a cancer treatment strategy.

We previously reported the overexpression of vimentin in the tumor vasculature^8^, a finding that was confirmed by others^20^. While overexpression of vimentin in aggressive tumors is well-known as it is the classical hallmark of EMT and associated with poor survival^13^, these features are attributed to intracellular functions of vimentin in tumor cells. Our current data show that extracellular endothelial vimentin is targetable in tumors regardless of tumor cell-intrinsic vimentin expression levels.

Active secretion of vimentin from (tumor) ECs, was not reported to date. Leaderless proteins can be secreted by pore-mediated translocation across the membrane (type I UPS), ABC transporter-based secretion (type II UPS), or autophagosome/lysosome/endosome-based secretion (type III). In addition, type IV unconventional secretion concerns proteins with a signal peptide that bypasses classical Golgi-mediated secretion^21^. e.g., IL-1β and FGF2 are externalized by these types of secretion involving multiple membranous structures, i.e., inflammasomes, autophagosomes, and secretory lysosomes, rather than by conventional Golgi- or ER-mediated externalization^22,23,39^. Through screening of a large repertoire of compounds that affect different types of UPS, we identified that vimentin is secreted by type III UPS mechanisms. It is believed that many inflammatory and angiogenesis mediators are externalized by non-conventional processes to enable them to exert additional functions during exceptional circumstances, such as tumor growth and inflammation^40^, as in general, these processes are stress-induced^21^. Detailed molecular mechanisms of vimentin secretion, however, remain to be unraveled as lysosomes, autophagosomes and endosomes can interact at different levels^21,23,24,41^.

The assembly and disassembly of vimentin intermediate filaments contribute to its highly dynamic nature, and the disassembly of filaments is the result of site-specific phosphorylation of serine residues in the N-terminal head domain of vimentin^42^. Although we did not directly observe the influence of perturbations of global phosphorylation on the secretion of vimentin from ECs, immunofluorescence studies show that the deposited extracellular vimentin is not filamentous. It remains to be investigated to what extent the extracellular fraction of vimentin is derived from phosphorylation and secretion, or from de novo synthesis, and whether or not this influences extracellular activities.

Furthermore, cellular stress and autophagy, e.g., during chronic inflammation and tumor progression, can cause citrullination of vimentin. This creates immunogenic epitopes that can give rise to autoantibodies or can be helpful in antitumor responses^43,44^. Regardless of possible posttranslational modifications (PTMs) in extracellular vimentin in vitro or in vivo, our data demonstrate functional effects of both application and (antibody-based) targeting of unmodified vimentin.

We here demonstrate that extracellular vimentin specifically interacts with and activates VEGFR2 and modulates VEGF signaling, increases VEGF receptor expression, and shares functional modes of action with VEGF. VEGF induces endothelial permeability, a.o. through direct interaction between VEGFR2 and VE-cadherin, resulting in transactivation of VE-cadherin and subsequent activation of β-catenin and internalization of VE-cadherin^45^. Our finding that extracellular vimentin can directly activate VEGFR2 places vimentin as an additional player in this process. Interestingly, extracellular vimentin has been reported to induce phosphorylation of β-catenin in colorectal cancer cells accompanied by activation of the Wnt pathway, although no cellular receptor was conclusively identified^15^.

Other putative cell surface receptors that interact with vimentin, which may play relevant roles in tumor angiogenesis and immune suppression, have been identified. These interactions may enhance or synergize with the here reported binding of vimentin to VEGFR2 and its consequent effects. For example, insulin-like growth factor 1 receptor (IGF1R), extensively involved in tumor angiogenesis^46^ was shown to be activated by the C-terminus of vimentin, thereby promoting axonal growth^47^, a process that shows resemblance to blood vessel formation. In addition, the hyaluronic acid-binding domain of CD44, an EC- and leukocyte adhesion receptor^48^, was demonstrated to interact with the N-terminus of vimentin^49^. Together with the observation that vimentin can bind P-selectin, also involved in EC-leukocyte interactions^50^, these findings indeed support a multifaceted modulatory role for extracellular vimentin in tumor angiogenesis and immunity.

In all, our data demonstrate that vimentin, like VEGF, (i) reduces VE-cadherin expression, cell-cell interactions and vascular integrity, (ii) supports tumor angiogenesis, and (iii) hampers antitumor immunity. It is therefore considered that extracellular vimentin is a master regulator of EC anergy, the phenomenon of tumor endothelial non-adhesiveness and unresponsiveness to inflammatory cytokines^3^. EC anergy was recently assigned the role of vascular immune checkpoint^5^. As such, inhibitors of angiogenesis can overcome EC anergy and potentiate immunotherapy, a concept that is currently in full development in the clinic^5^. Also, vimentin potentiates the expression of endothelial PD-L1, leading to immune exhaustion, and vaccination against vimentin was demonstrated to suppress tumor endothelial PD-L1 expression.

**Fig. 7:**
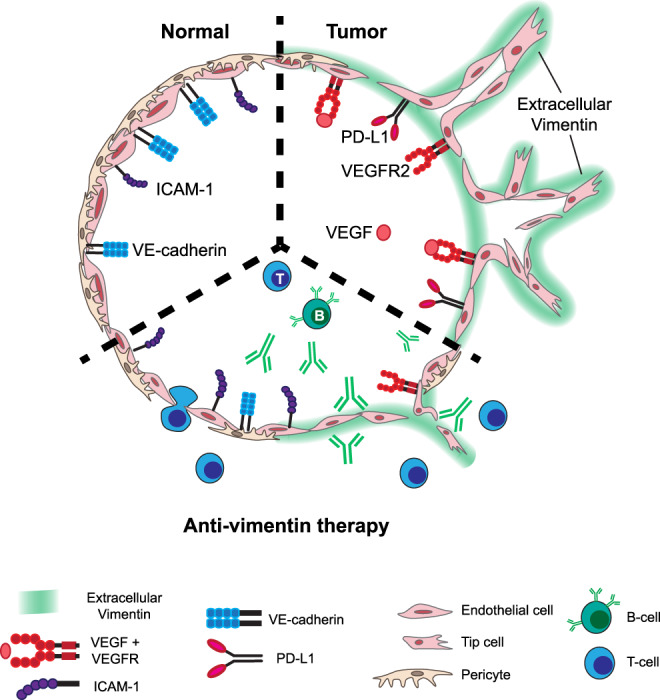
Anti-angiogenic immunotherapy targeting extracellular vimentin. Secreted, extracellular vimentin in the tumor vasculature contributes to the upregulation of VEGFR2 and PD-L1 expression and suppresses ICAM1 expression. Anti-vimentin immunotherapy restores ICAM1 expression, enhances immune cell infiltration, and suppresses PD-L1 expression.

Vaccination against vimentin resulted in reduced tumor growth explained by the induction of a robust vimentin-specific humoral response, altered expression of leukocyte adhesion molecules, and a notable switch in the intratumoral immune cell repertoire. Specifically, tumors derived from vimentin-immunized mice were characterized by higher frequencies of professional antigen-presenting cells, namely dendritic cells (DCs). Although DCs constitute only a small fraction of the total pool of tumor-infiltrating lymphocytes, they play a pivotal role in terms of orchestrating local immune activation and subsequent recruitment of other immune effector cells^51^. Moreover, tumor-infiltrating DCs are highly conserved across solid human cancers^52,53^, their maturation status defines antigen-specific T-cell avidity^54^ and they are associated with positive prognosis^55^. Besides the elevated number of DCs, we noted a shift from immature myeloid Cd11b^+^F4/80^+^Ly6C^+^ cells towards differentiated macrophages in the vimentin-vaccinated group. This alteration might have direct implications for the obtained tumor regression phenotype, since Cd11b^+^F4/80^+^Ly6C^+^ cells exert immune-suppressive functions and account for increased tumor growth and metastasis formation. Additionally, vaccination against vimentin decreased the rate of M-MDSCs, which constitute the most well-characterized immune-suppressive cell type found in tumors^56^. M-MDSCs can downregulate antitumor immune responses mediated by NK and T cells by using nitric oxide (NO), immunosuppressive cytokines (IL-10 and TGFβ), and high PD-L1 expression^57^. Indeed, we observed a reciprocal relationship between infiltration rates of suppressive M-MDSCs and stimulatory NK and NKT cells in the tumors of mice. Also, Pd-1 expression on NKT cells, as well as IL-10 cytokine secretion tended to be lower in tumors of vimentin-vaccinated mice. Alternatively, the improved levels of macrophage differentiation and NK cell recruitment could also be coupled to the interaction between their Fc gamma receptors and the anti-vimentin antibodies that were induced upon vaccination, contributing to antibody-dependent cellular phagocytosis and antibody-dependent cellular cytotoxicity, respectively^58,59^. In total, vaccination against extracellular vimentin boosts antitumor immunity and favors the establishment of a less immune-suppressive tumor microenvironment. Together, our results suggest that a targeting strategy against extracellular vimentin will inhibit angiogenesis and revert immune suppression, making it an attractive therapeutic target (Fig. 7).

While monoclonal antibodies have become serious therapeutic players, a polyclonal response evoked by vaccination is potentially much more effective. A broader polyclonal reactivity better blocks the extracellular functions of vimentin. Induction of polyclonal antibody responses is usually also more efficient at inducing antibody- and complement-dependent cytotoxicity^10^, compromising the tumor vasculature while at the same time enhancing antitumor immunity. Additional advantages of vaccination over the use of monoclonal antibodies are (i) higher penetration capacity of endogenous antibodies, (ii) possibility for multi-epitope or multi-target approaches, (iii) long-term efficacy, (iv) low level of invasiveness, and (v) excellent cost-effectiveness. Preclinical studies in rodents, as well as the efficacy study in client-owned dogs with spontaneous bladder cancer, show that vaccination against extracellular vimentin is safe, emphasizing the specificity of extracellular vimentin for tumor angiogenesis. We foresee that a safe and effective vaccination strategy, as presented here, can be readily applied in a clinical setting, as we have previously shown with vaccinations against a truncated form of VEGF^60^.

In conclusion, extracellular vimentin secreted by tumor ECs is a crucial player in tumor angiogenesis, immune infiltration, and immune suppression. This finding lends multiple dimensions to the effects of targeting vimentin is an anticancer setting, while a vaccination approach offers a safe and effective strategy.

## Methods

### Ethics statement

All experiments conducted in this study were approved by local regulatory boards and complied with national and international regulations. Details are included in the respective sections below.

### Cell culture

HUVEC were freshly isolated from umbilical cords (approved under the “Code Goed Gebruik” as defined by FEDERA and COREON under the Dutch National Medical Ethics body (Amsterdam UMC medical ethical committee waiver: W12–167#12.17.096); obtained from the Department of Obstetrics and Gynecology, Amsterdam UMC, Amsterdam, The Netherlands) and maintained in RPMI supplemented with 10% bovine calf serum (NBCS) (Sigma-Aldrich, St. Louis, USA) and 10% human serum^61^. PBMCs were purchased from Sanquin, Amsterdam, The Netherlands.

RF24 (immortalized human vascular ECs; gift^62^), HMEC-1 (immortalized human vascular ECs; ATCC CRL-3243)^63^, and Jurkat (immortalized human T-lymphocytes; ATCC TIB-152) were maintained in RPMI cell culture medium supplemented with 1% of antibiotics (penicillin/streptomycin, Life Technologies, Carlsbad, California, USA) and 10% NBCS. Tumor cell lines 786-O (human renal cell carcinoma; ATCC CRL-1932)^64^, MDA-MB-231 (human breast carcinoma; ATCC CRM-HTB-26)^65^, A2780 (human ovarian carcinoma; ECACC 93112519)^66^, HCT116 (human colorectal carcinoma; ATCC CCL-247)^67^ were maintained in DMEM supplemented with 1% of antibiotics and 10% NBCS, as were the murine cell lines B16F10 (mouse melanoma; ATCC CRL-6475)^68^, SVEC 4-10 (mouse ECs; ATCC CRL-2181)^69^ and CT26 (mouse colorectal carcinoma; ATCC CRL-2638)^70^. Cells were originally obtained from ATCC or ECACC (A2780) or were a gift (RF24), and were routinely tested for the absence of mycoplasma. All cell assays as reported were performed on 3 to 5 independent passages or donors.

### Compounds and reagents

Compounds used to interfere with secretion pathways (Fig. 1) are detailed in Supplementary Table 1. Recombinant vimentin, purified from insect cells, was obtained from SinoBiologicals. VEGF refers to recombinant VEGFA165, obtained from Preprotech. Kits and essential reagents are detailed in Supplementary Table 3. Antibodies used in in vitro and in vivo assays, and for detection of proteins by immunofluorescence, immunoblotting, or single-color flow cytometry and ELISA are detailed in Supplementary Table 4. Antibodies were dialyzed against 0.9% NaCl to remove traces of azide, for application in vitro and in vivo. Antibodies used in immunohistochemical stainings, along with protocol details, are presented in Supplementary Table 5. Antibody panels used for immune profiling by flow cytometry are detailed in Supplementary Table 6. qPCR primers are listed in Supplementary Table 7. Details of public data sets are listed in Supplementary Table 8.

### EC viability and proliferation assay

ECs (5 × 10^3^ cells/well for HUVEC, 1 × 10^4^ cells/well for RF24) were seeded in 0.2% gelatin-coated 96-well cell culture plates^71^. Cells were treated as indicated. Cell viability was assessed using the CellTiter-Glo^®^ luminescent Cell Viability Assay (Promega) according to the manufacturer’s instructions.

### EC migration assay and sprouting assay

EC migration was performed on confluent monolayers in gelatin-coated 96-well cell culture plates. A wound of approximately 300 µm wide was made using a guided 96-well pin tool (Peira, Turnhout, Belgium). Wells were washed to remove cell debris and the medium was added. Images were captured with a Leica DMI3000 microscope (Leica, Rijswijk, The Netherlands) with Universal Grab 6.3 software (DCILabs, Keerbergen, Belgium), at time points *T* = 0 h to *T* = 8 h^61^. Where indicated, wound closure (μm^2^) was expressed as a percentage of control wells.

EC spheroids were created using the hanging drop technology^72^. Briefly, 1000 HUVEC were resuspended in 25 μl aliquots containing medium supplemented with 20% methocel (Sigma-Aldrich), and drops were incubated on the inverted lid of a PBS-filled petri dish. Twenty-four hours later, the drops were gently harvested and embedded in type I bovine collagen gel (2 mg/ml, Advanced BioMatrix), at approximately 20 spheroids per well in 8-well microscope slides (Ibidi). After solidification, medium and compounds were added on top of the gel. Quantification of sprouting was performed using a semi-automatic ImageJ-based macro^73^ on 5–25 spheroids per condition. Cells were treated as indicated.

### Matrigel assay

Specialized cell culture slides (µ-Slide Angiogenesis, Ibidi) were filled with 10 µl of Matrigel (Corning) which was allowed to solidify. In all, 5 × 10^3^ HUVEC were added in a total volume of 50 µl medium, and treated as indicated. Tube formation was monitored for *T* = 8 to *T* = 16 h. Images were analyzed with the Angiogenesis Analyzer plugin^74^ in ImageJ v1.50i.

### Cell cycle analysis

Analysis of cellular DNA content using propidium iodide was performed using flow cytometry^75^. Cells were seeded in a 24-well plate at a density of 2–4 × 10^4^ cells/well and incubated for 24 h prior to treatment as indicated. Cells were harvested by trypsinization and fixated in 70% ethanol for 2 h at −20 °C. Cell pellets were then resuspended in DNA extraction buffer (90 parts 0.05 M Na_2_HPO_4_, 10 parts 0.025 M citric acid, 1 part 10% Triton-X100, pH 7.4) and incubated for 20 min at 37 °C. Propidium iodide (PI, 20 μg/ml; Life Technologies) was added, and cells were analyzed with a FACSCalibur flow cytometer (BD Biosciences). DNA content was quantified with CellQuest Pro software (BD Biosciences).

### siRNA transfection

HUVEC (1 × 10^4^) were seeded in 96-well tissue culture plates that were coated with gelatin and where 50–200 nM siRNA (Eurogentec, Liege, Belgium) and 1.5 µl transfection reagent (HiPerfect; Qiagen) were complexed for 20 min at RT. Cells were processed for downstream analysis 48–72 hr later^71^.

### Lymphocyte adhesion and transmigration assays

HUVEC (1 × 10^4^) or RF24 (2 × 10^4^) were seeded in gelatin-coated 96-well tissue culture plates and grown to confluence. Cells were pretreated with 20 ng/ml TNFα (Preprotech) for 2 h, followed by the addition of 1 × 10^5^ Jurkat cells with or without recombinant vimentin. Plates were incubated for another 2 h to enable stable interactions between Jurkat and ECs. Culture medium and unbound cells were aspirated, followed by four washes by PBS. Images were captured using a Leica DMIL microscope and bound Jurkat cells were manually counted in five imaged fields per well.

For transmigration assays, HUVEC (3 × 10^4^) were seeded in a 3 µm pore transwell inset in 24-wells plates (Costar; Merck) and grown for 24 h to reach confluence. Recombinant vimentin and/or VEGF (Preprotech) were added to the bottom compartment of the transwell system, and calcein-AM (Life Technologies) labeled human PBMCs (2 × 10^5^) were added to the top compartment. Plates were incubated for 16 h and transmigrated cells in the bottom compartment were counted using a Coulter counter. In parallel, 500 µg/ml 70 kDa FITC-Dextran (Sigma-Aldrich) was added to the upper compartment in the presence or absence of vimentin and/or VEGF, and the medium in the lower compartment was sampled for fluorescence on a BioTek Synergy HT microplate reader after 1 hr. All data were normalized to untreated controls.

### Chorioallantoic membrane of the chicken embryo (CAM) assay

Detailed methods for growth, handling, and treatments of the eggs have been described elsewhere^76,77^. Briefly, fertilized chicken eggs were incubated for 3 days with automatic rotation, before a pinhole was created in the shell. Eggs were incubated standing up for the remainder of the experiment. Effects of recombinant vimentin and anti-vimentin antibodies in the developmental chicken embryo CAM assay were assessed via topical administration on the CAM on embryo development day (EDD) 7 and 8 at the indicated concentrations. Vasculature was visualized and analyzed on incubation day 9^76,77^.

Visudyne^®^*-*Photodynamic therapy (PDT)^29^ was performed on EDD11. Within PDT-treated areas, 20 μl anti-vimentin antibodies (10 μg/ml) were administered topically twice, immediately after PDT and 24 h later. Quantification based on the fluorescence angiographies was performed on EDD13^76^.

Tumor growth experiments on the CAM employed tumor cells (HCT116, MDA-MB-231, A2780, 786-O; 1–5 × 10^6^) mixed with Matrigel and placed onto a denuded area of the CAM on EDD8. Tumors were allowed to grow until EDD12 prior to daily treatment as indicated until EDD17^71,78^.

For imaging, anti-vimentin antibody RV202 was biotinylated according to the manufacturer’s guidelines (Roche), and pre-incubated with Alexa488-labeled streptavidin (DAKO), prior to i.v. injection in an A2780 xenografted CAM. After circulation for up to 1 hr, CAMs were imaged with an epi-fluorescence microscope (Nikon Eclipse E600 FN, Japan)^76^.

### Flow cytometry

Surgical material (tumors and adjacent normal tissues) was obtained after the informed consent of the patient, and sent for routine pathological evaluation and FFPE processing at the Department of Pathology, Maastricht University Medical Center, Maastricht. Following macroscopic evaluation and dissection, part of the freshly resected tissue was used for flow cytometry and qPCR as described further below. Flow cytometry for the detection of vimentin in tissue-isolated EC was performed by double labeling of EC in single-cell suspensions from human tumor and normal colon samples using a combination of PE-labeled mouse anti-human CD31 (1:25, DAKO) and rabbit anti-human vimentin (1:50, Abcam)^8^. Cultured ECs were trypsinized, fixated with 1% PFA, and stained with antibodies as indicated. FITC-labeled secondary antibodies were used for fluorescent detection. All antibody incubations were performed in PBS/0.5% BSA for 1 h at RT. Cells were analyzed on a FACSCalibur (BD Biosciences) and data were analyzed using CellQuest Pro software (BD Biosciences).

For profiling of immune cell subsets, B16F10 tumors of control and vimentin-vaccinated mice were excised, mechanically dissociated with scissors, and subsequently incubated in 5 ml of digestion mix containing 1 mg/ml collagenase IV (Sigma), 30 U/ml DNAse I type II (Sigma) and 0.1 mg/ml hyaluronidase type V (Sigma) in RPMI for 25 min at 37 °C under gentle agitation. Following quenching of enzymatic activity by addition of RPMI, cell suspensions were filtered through a 70 µm cell strainer, pelleted, and resuspended in 5 ml RPMI supplemented with 10% FCS, 1% penicillin/streptomycin, and 50 µM 2-mercaptoethanol. Cells were subsequently layered on Ficoll and interphase cells following centrifugation were carefully transferred to fresh tubes. Cells were counted and diluted to 10*10^6^ cells per ml. One million cells were stained for analysis of immune cell subsets, details of the antibodies are shown in Supplementary Table 6.

In more detail, cells were transferred to a V-bottom 96-well plate (Greiner Bio-One), washed once with PBS, and resuspended in TruStain Fc blocking solution (BioLegend) for 10 min at RT. Afterwards, cells were incubated with primary antibodies diluted in PBS for 20 min on ice. Cells were washed once with PBS and fixed with 4% paraformaldehyde for 15 min on ice. After fixation, cells were washed once with PBS and permeabilized using the intracellular staining permeabilization wash buffer (BioLegend). Cell suspensions were then incubated with antibodies directed at intracellular antigens, in the above-mentioned buffer for 30 min at room temperature. Cells were washed twice with the permeabilization wash buffer, resuspended in 100 μl PBS and transferred to FACS tubes. Cell suspensions were analyzed on a Fortessa LSR (BD Biosciences) and data were analyzed using FlowJo software (v10; BD Biosciences).

Gating details are shown in Supplementary Figs. 8 and 9. Primarily, cell suspensions were pre-gated on single live Cd45+ cells, followed by further subclassification based on marker expression as denoted, to obtain population statistics (population percentage, mean and median fluorescence intensity). For the visualization of the data in tSNE plots, samples were concatenated based on single live Cd45+ cells, and analyzed with the tSNE functionality in FlowJo v10, under default settings (1000 iterations, perplexity 30, Barnes-Hut algorithm). Gated populations were subsequently colored as indicated.

Analysis of soluble cytokines was performed using the LegendPlex mouse Inflammation panel (BioLegend), according to the manufacturers’ instructions. Briefly, B16F10 tumors from control and vimentin-vaccinated mice were mechanically dissociated and incubated in PBS with protease inhibitor cocktail (Roche) and 1 mM PMSF (Sigma-Aldrich) for 1 h at 4 h at 37°C on a Vortex-Genie 2 at 600 rpm. Samples were centrifuged at 12,000 × *g* for 10 min and the supernatant was used to determine total protein concentrations in the secretome with a BCA assay (Thermo Fischer Scientific). Samples were diluted to 2 mg/ml input in the bead-based assay that was analyzed on a FACSCalibur (BD Biosciences); data were analyzed using Legendplex Data Analysis Software Suite.

### qPCR

Isolation of total RNA (RNeasy mini; Qiagen), complementary DNA synthesis (iScript; Bio-Rad), and qPCR (SYBR green; Bio-Rad) were performed according to the manufacturers’ instructions. Briefly, ECs were isolated from freshly resected colorectal tumors and patient-matched normal colon^8,79^, cultured ECs were trypsinized and washed with PBS, and frozen tumors were homogenized in RLT buffer prior to RNA isolation.

CAMs and CAM tumors were excised, fixated in zinc-fixative solution^80^, and stored before RNA isolation with Trizol (Life Technologies) or processing for immunohistochemistry. Primers that distinguish between human and chicken mRNAs were used to profile vimentin expression in the CAM vessels, and were designed according to previously published guidelines^81^.

Assays were run on a Bio-Rad CFX96 thermal cycler and analyzed using CFX Manager software v3.1^71^. In vitro assays were performed on 3 to 5 independent passages (HMEC-1) or donors (HUVEC), and analyzed in up to 3 independent experiments. Of thus generated 9 to 15 analyses, only samples showing appropriate melting curves and relevant Ct values were included in subsequent analysis. Relative gene expression was calculated with the 2^−ΔΔCT^ method and expressed as (transformed) percentage of control conditions where indicated. Primers are listed in Supplementary Table 7.

### ELISA for vimentin secretion

Secreted vimentin was detected in the conditioned medium (CM) of ECs by coating 50 µl of CM in ELISA microplates (Nunc). Alternatively, the secretome of B16F10 tumors was used. For estimation of concentrations of secreted vimentin, CM or secretome was stepwise diluted in PBS and assayed in parallel with a standard curve of recombinant vimentin. For evaluation of compounds affecting the secretion of vimentin, cells were treated as described above with the three highest concentrations of compounds that did not affect cell viability, and CM was analyzed in relation to untreated or solvent-treated cells.

Following coating in microplates, plates were blocked with 4% non-fat dry milk in PBS, and wells were subsequently incubated with primary antibody (V9; DAKO), biotinylated goat-anti-mouse Ig (DAKO), and streptavidin-HRP (DAKO), as detailed in Supplementary Table 4.

All incubations were performed for 1 h at 37 °C and in between steps plates were washed 3× with PBS/0.1%Tween-20. All incubation volumes were 50 µl, except for the blocking (4% non-fat dry milk (ChemCruz) in PBS) which was 150 µl. Color development was performed with standard TMB solution (Sigma-Aldrich) and stopped with 2 N H_2_SO_4_. Plates were analyzed with a Biotek Synergy HT microplate reader (Biotek), for OD at 450 nm, along with a background reference at 540 nm.

### Western blotting and proteomics analysis

HUVEC were cultured to near confluence in replicate cell culture dishes. For the last 6 hours, cells were incubated with a serum-free medium after washing with PBS to generate BSA-free secretome. Conditioned medium was collected and concentrated 10 times on a spin column (Millipore). HUVEC were washed with PBS and detached with citric saline cell detachment solution (135 mM KCl, 15 mM sodium citrate) and pelleted for lysis. After verification that all cells had detached, PBS was added to the ECM deposit in the plates, scraped vigorously with a cell scraper, and collected.

Protein concentrations were evaluated using a micro BCA protein assay (Thermo Fischer Scientific). Fifteen to 50 µg of proteins per condition was separated on 4–12% polyacrylamide gels (Invitrogen) and transferred to a polyvinylidene difluoride membrane. Odyssey blocking buffer (LI-COR Biosciences) was used to block membranes and following incubation with primary and infrared-dye secondary antibodies (LI-COR). Images were obtained with the LI-COR Odyssey CLx scanner at one default exposure setting.

For standard proteomics analysis of the content of the different cell fractions, the samples were processed according to established protocols^82^, and deposited in the PRIDE repository under accession number PXD024426. Briefly, following SDS-PAGE, sections were cut from the gel, and slices were digested with trypsin prior to LC-MS/MS. Peptide counts were aggregated to proteins and normalized to the total count in each sample. Enrichment for a particular fraction was determined using a modified binomial test^82^. Peptide coverage for vimentin in each fraction was retrieved from the raw data and plotted as a count profile which reflects both the propensity to be analyzed (presence and frequency of lysines that are targeted by trypsin and determine inclusion in the analysis) as well as the distribution of the protein fragments of vimentin present in each fraction to determine any differences.

Proteins enriched in the extracellular fractions over the total protein lysate were analyzed with secretomeP and proteinside databases for the presence of signal sequences and odds of secretion. Relevant subsets of proteins were subject to interaction analysis using STRING and functional enrichment using Enrichr. Protein-protein interactions were visualized using Cytoscape v3.7.2.

### VEGFR2 phosphorylation and binding

Cells were cultured in 10 cm dishes under routine conditions, until near confluence. Plates were drained and cells were washed gently with 5 ml PBS. Next, 5 ml medium containing recombinant vimentin and/or VEGF at the indicated concentrations was added and cells were incubated for 15 min. The medium was drained, and cells were washed with PBS and placed on ice immediately. Cells were lysed, and concentrations were determined with a BCA assay (Thermo Fischer Scientific) and normalized to 500 µg/ml. Samples were analyzed according to the DuoSet ELISA VEGFR2 (R&D systems) instructions for quantification of VEGFR2 receptor phosphorylation status.

Surface Plasmon Resonance (SPR) biosensor assays were carried out using Biacore T200 (GE Healthcare) with CM5 sensor chips (Cytiva). VEGFR2 receptor (VEGFR2-Fc; BioLegend) at a concentration ~10 µg/ml in 10 mM acetate buffer pH 4 was immobilized at the density of ~900 RU using the amine-coupling kit (Cytiva) according to the manufacturer’s protocol at a flowrate of 5 µl/min. Concentration series of recombinant human vimentin and VEGF diluted in the running buffer (PBS, 0.05% Tween-20, pH 7.4) were injected over the sensor chip surface at 30 µl/min flowrate, at 25 °C for 180 sec. Dissociation of formed complexes was followed for 240 sec after the end of an injection. After each cycle, the chip surface was regenerated by 60 s injections of 1 M ethanolamine-HCl, pH 8.5. Obtained sensorgrams were double referenced.

For ELISA-based detection of interaction, recombinant human vimentin, VEGF, or BSA (2 µg/ml) were coated in ELISA plates, followed by blocking with 4% non-fat dry milk in PBS. VEGFR2-Fc was added in a concentration range from 10 ng/ml to 5 µg/ml and detected with biotinylated goat-anti-human Fc antibody (MP Biomedicals) in combination with streptavidin-HRP, essentially as described above.

Alternatively, recombinant vimentin, recombinant VEGFR2, or BSA were spotted on nitrocellulose membranes (Sigma-Aldrich) in a gridwise fashion. Spotblots were blocked with 4% blocking reagent (Bio-Rad) in PBS, and subsequently incubated with recombinant vimentin, recombinant VEGFR2, or BSA. Anti-vimentin antibodies or anti-VEGFR2 antibodies (1:100 in 1% BSA/PBS) were used to detect proteins interacting with the immobilized proteins. Detection was done with biotinylated goat-anti-mouse IgG and streptavidin-HRP. Blots were developed with ECL Pico Plus reagent (Pierce).

### Immunofluorescence

For immunofluorescence studies, cells were seeded in 96-well plates and grown overnight unless otherwise indicated. Briefly, cells were washed, and blocked in 1% BSA in PBS. Primary antibodies were added in 0.5% BSA, followed by washes with PBS. Primary antibodies were detected with biotinylated rabbit- or goat-anti-mouse IgG and streptavidin-Alexa488. For detection of intracellular vimentin, cells were fixated with 1% PFA in PBS and permeabilized with 0.1% Triton-X100 in PBS prior to blocking. For detection of vimentin in ECM deposit, cells were either removed with different cell removal agents as indicated, or left present in the plate, but without any fixation. Antibody incubations were performed for 45 min at RT for fixated cells and for 30 min on ice with live cells. Stained live cells were post-fixated and permeabilized, and nuclei and F-actin were subsequently stained with DAPI (Sigma) and Phalloidin-TRITC (Life Technologies), respectively, where applicable.

Images were captured using a Leica DMIL microscope with a fluorescence unit in combination with an FC345Fx camera, with a ×20 objective. High-resolution microscopy was performed after growing HUVEC in eight-well ibiTreat chamber slides (Ibidi), and images were analyzed on a STED system (Leica Microsystems, at AO|2 M facility Amsterdam UMC) or a Leica TCS SP5 Confocal system (Leica Microsystems at NKI Amsterdam)^83^.

Images were analyzed using Leica Application Suite v4.13.10 (Leica), and were, where necessary for presentation in the figures, merged to construct RGB images and post-processed using Adobe Photoshop CS6 to enhance color contrast. Any modifications were applied to whole images only.

### Immunohistochemistry

Normal and tumor tissues were paraffin-embedded and sectioned (5 µm) with a Leica RM 2135 microtome. CAM and CAM tumors were pre-fixated in zinc fixative prior to paraffin embedding and sectioning. Sections were dried overnight at 37 °C, placed at 60 °C for 1 h, and baked for 10 min at 56 °C before deparaffinization with xylene (VWR International) followed by 100% (Nedalco), 96%, and 70% ethanol and rehydration in phosphate-buffered saline (PBS). Alternatively, tumors were snap-frozen in liquid nitrogen and sectioned with a Leica CM1850 UV research cryostat.

Protocol details and antibodies are presented in Supplementary Table 5. In general, after treatment with hydrogen peroxide (Hydrogen peroxide 30%, BDH Prolabo, VWR International) in PBS or methanol for 15 min at RT, antigen retrieval was performed in a microwave oven or autoclave. After cooling down, sections were washed in PBS and blocked with BSA or serum diluted in PBS for 1 h at RT and incubated with primary antibody diluted in 0.5% BSA/PBS overnight at 4 °C. The next day, tissue sections were incubated with biotinylated secondary antibodies and streptavidin-HRP or HRP-labeled secondary antibodies for 45 min at RT. For detection of anti-vimentin treatment antibodies in CAM xenografts, only secondary detection was performed. Sections were washed 3 × 3 min in PBS in between antibody incubations. Color development was done using 3,3′-diaminobenzidine tetrahydrochloride hydrate (DAB) staining (Sigma-Aldrich). Sections were counterstained with Mayer’s hematoxylin (Klinipath) for 30 s and the reaction was stopped under running tap water for 10 min and mounted with Quick D mounting medium (Klinipath).

For morphological detection of immune cells and histological evaluation of tissues, frozen or deparaffinized sections were dipped in diluted Mayer’s Hematoxylin (Klinipath) (1:4 dilution in 5 mM sodium citrate buffer pH 6.0). After a rinse under flowing tap water for 5 min, sections were stained with 0.2% eosin Y solution (J.T. Baker, Avantor Performance Materials) for 30 s. Sections were dehydrated with two changes of 70% ethanol, three changes of 96% ethanol, 100% ethanol for 5 min, and xylene for 2 min. Consecutively, sections were mounted with Quick D mounting medium (Klinipath).

Only viable tumor tissue was used for analysis. The number of vessels and immune cells was counted or scored manually based on the morphology of HE stained sections or antibody stainings (Cd31, Cd3, F4/80). Up to 5 fields/tumor at 200× magnification (HPF 0.25 µm^2^) were counted. Icam1 staining was quantified as the percentage area above the threshold following processing with the Color Deconvolution plugin v1.8B in ImageJ. Pd-l1 staining was manually scored for the staining intensity of perfused vessels. Where relevant, images were taken with an Olympus BX50F microscope equipped with a CMEX5 camera (Euromex), and captured using ImageFocus4 (Euromex).

### In silico analysis

Images of different tumor types and normal tissues stained for vimentin were retrieved from the Human Protein Atlas^84^. For correlation analysis, five different colorectal cancer data sets with Affymetrix gene expression data (specified in Supplementary Table 8) were used and analyzed with R2 for other genes correlating with vimentin expression. Overrepresentation analysis for functions and pathways was performed using Webgestalt.

NCBI Gene expression omnibus (GEO) was searched for data sets containing gene expression analysis of isolated ECs from the tumor and normal tissues. Data were processed in R Studio (2021.09.01, build 372) using R version 4.1.2, and analyzed for vimentin expression.

In silico analysis of (immune) cell subsets based on bulk RNA expression was performed using published methods and tools. The murine Microenvironment Cell population counter (mMCP-counter)^30^ was applied for analysis of RNAseq data of B16F10 tumors of control and vimentin-vaccinated mice. In addition, GEO data sets (Supplementary Table 8) were obtained and normalized expression values were used to divide data sets into high and low vimentin expressing samples, and data were input in Cibersort^32^ for in silico evaluation of immune infiltrate.

### Vaccine production and purification

The recombinant vaccine proteins were produced and purified based on established protocols, with modifications^10,70^. Murine (NM_011701) and dog (NM_001287023.1) vimentin protein-coding sequences (either alone or in frame with thioredoxin (TRX) or truncated thioredoxin (TRXtr) - hereafter for both mouse and dog referred to as (TRXtr-)Vimentin) - were cloned in the pET21a expression vector which was transformed into *E.coli* BL21 (Novagen; Merck Millipore) for recombinant protein expression. The pET21a-TRX plasmid was transformed into Rosetta gami (DE3) (Novagen). Overnight cultures were diluted 1:3 and grown until an optical density 600 nm (OD600) of 0.5 was reached. Protein expression was induced with 1 mM isopropyl b-D-1-thiogalactopryanoside (IPTG, Invitrogen, Life Technologies) at 37 °C for 4 h. Bacteria were harvested by centrifugation and bacterial pellets were dissolved in 5 M urea (TRX) (Acros Organics/Thermo Fisher Scientific) or 2 M urea, 20% glycerol, 0.1 µM EDTA, 1% Triton-X100 (TRXtr-Vimentin). The proteins were released by sonication (amplitude 22–26 microns using a Soniprep 150 MSE) on ice, 12 times for 30 s with breaks of 30 s (TRX) and 15 times for 20 s with breaks of 30 s (TRXtr-Vimentin). Thereafter for TRX, 1 ml 50% Ni-NTA agarose slurry (Qiagen) was mixed with 25 ml supernatant (originating from 500 ml bacteria culture) and 10 mM imidazole (J.T.Baker, Avantor Performance Materials), to reduce non-specific binding of background proteins to the nickel (Ni) agarose, and incubated “end-over-end” at 4 °C overnight. For TRXtr-Vimentin 1 mM PMSF (Sigma-Aldrich) was added to the supernatant after sonication and 200 µl 50% Ni-NTA agarose slurry was mixed with 5 ml supernatant (originating from 50 ml bacteria culture). The next day the agarose beads were spun down and the supernatant was kept and frozen at -20 °C overnight. Hereafter again 300 µl 50% Ni-NTA agarose slurry was added to the supernatant (originating from 100 ml bacteria culture) and incubated “end-over-end” at 4 °C overnight. After centrifugation, the agarose beads were washed with 250 ml wash buffer containing PBS pH 7.0/1 M NaCl /0.05% Tween-20. An additional washing step with PBS was performed to remove the detergent. Then, the beads were transferred to a 1 ml syringe (BD Biosciences) with a glass filter (Sartorius Stedim Biotech) and washed again with PBS. The column of the TRX protein was washed with 10 fractions of 500 μl 20 mM imidazole, dissolved in 20 mM Tris pH 8.0/0.1 M NaCl, and eluted with four 500 μl fractions of 200 mM imidazole, dissolved in 0.2 M Tris pH 8.0/0.1 M NaCl. For TRXtr-Vimentin the column was washed with four 150 µl fractions of 200 mM imidazole dissolved in 0.2 M Tris pH 8.0/0.1 M NaCl and the protein was eluted with four 150 µl fractions of buffer E (100 mM NaH_2_PO_4_, 10 mM Tris, 8 M urea, pH 4.5). The protein content of the separate fractions was determined by SDS-PAGE and colloidal Coomassie brilliant blue G250 staining (Fischer). Fractions containing most protein were pooled and dialyzed stepwise towards 2 M urea/PBS ((TRXtr-)Vimentin) or towards PBS (TRX). TRXtr-conjugated proteins were used for the vaccination of mice and dogs, whereas unconjugated vimentin was used in ELISA for the determination of antibody levels. Thioredoxin (TRX) was used for the vaccination of control mice.

### Mouse experiments

Animal experiments were approved by the local Animal Ethics Committee (DEC) of the VU University and the national Central Animal Experiments Committee (CCD) (reg. no. DEC AngL14-01, CCDAVD114002016576, and CCDAVD1140020173104, including VUmc animal welfare body approved work protocols 576-ANG19-05, 576-ANG21-08, 576-ANG17-03, 576-ANG17-01, and 3104-ANG19-03). The maximum allowed tumor growth was 2 cm^3^. At the end of the experiment, mice were monitored (including tumor measurement) daily. When the maximum allowed tumor growth was exceeded - which was only the case in control groups at the last stage of the experiment - the animal was terminated. In case the maximum tumor volume was exceeded, the welfare of the animal was not compromised, and the appearance and activity were still normal.

For all experiments, 8-week old female C57BL/6 J (*C57BL/6OlaHsd*) mice or BALB/c mice (*BALB/cOlaHsd)* (Envigo, Horst, The Netherlands) (5–10 mice/treatment group) were allowed to acclimatize 2 weeks, housed at ambient temperature (20-24^o^C) and humidity (45–65%), with a 12/12 h light–dark cycle and fed ad libitum. Mice were immunized four times with an interval period of 2 weeks. Each vaccine emulsion (100 μl per mouse, 50 μl per groin) contained 20 μg TRX (control group) or 90 μg TRX(tr)-Vimentin in a volume of 50 μl mixed with 50 μl Freund’s complete adjuvant (F-5881, Sigma-Aldrich) (ratio 1:1, aqueous phase: oil phase) for the priming immunization and Freund’s incomplete adjuvant (F-5506, Sigma-Aldrich) for booster immunizations. Emulsions were mixed for 30 min on a Vortex Genie 2 (Fisher Scientific) at full speed. Two weeks after the last immunizations with TRX and TRXtr-Vimentin, 1 × 10^5^ B16F10 melanoma cells were inoculated subcutaneously in the left flank of C57BL/6 mice in a total volume of 100 μl (10% culture medium/PBS). For the CT26 model 2 × 10^5^ CT26 colon carcinoma cells were inoculated in the left flank of BALB/c mice, immunized with TRX, TRX-Vimentin, or TRXtr-Vimentin. Blood samples were taken from the tail vein 1 week after each immunization, 1 week after tumor cell injection, and at the end of the experiment. Tumor growth was measured by calipers. Tumor volume was calculated by the formula: width^2^ × length × π/6. At the end of the experiment, mice were euthanized and tumors and organs were removed and stored in 1% PFA/PBS overnight and consecutively paraffin-embedded, or frozen. Alternatively, fresh tissues were processed as described above for cellular immunoprofiling and cytokine analysis.

For the passive immunization experiments, ~8-week-old female C57BL/6 mice (*n* = 10/group) were inoculated in the left flank with B16F10 melanoma as described above. After palpable tumors were present (~50 mm^3^), mice were randomized and treatment started with antibody injections every 3 days intraperitoneally as previously described^8^.

For evaluation of wound healing, mice (C57BL/6) received three vaccinations with TRXtr-Vimentin (*n* = 5) or TRX (*n* = 5) as described above. Prior to the surgical procedure, per-operative analgesia buprenorphine 0.1 mg/kg body weight (Temgesic, Indivior Europe) was administered subcutaneously. During all procedures, mice were anesthetized with 2.5% isoflurane. The skin of the mouse was depilated with crème (Veet) and a full-thickness wound of 8 mm diameter was made on the back of the mouse with a biopsy punch (Kai Medical), and closure of the wounds was monitored over time. Wounds were protected from dirt with Cavilon no-sting barrier spray (3M). After surgery, the analgesic carprofen 0.042 mg/ml (Rimadyl; Zoetis) was given in the drinking water for a period of 1–2 days. The wound area was calculated with the formula π *(diameter/2)^2^.

To address the safety of prolonged exposure to high antibody titers against vimentin, control vaccinated (TRX, *n* = 5) and TRXtr-Vimentin (*n* = 5) vaccinated mice were included in the study for 45 weeks. Approximately 8-week-old female C57BL/6 mice were immunized three times with an interval period of 2 weeks as described above. Blood samples were taken from the tail vein 1 week after each immunization. During the rest of the follow-up period, monthly blood samples were taken. When antibody levels dropped below 50% of the levels after the third vaccination mice were revaccinated. In addition, the body weight of the mice was monitored regularly during the whole study period. At the end of the experiment, mice were euthanized and organs were removed, stored in 1% PFA/PBS overnight, and paraffin-embedded.

### ELISA for anti-vimentin antibody response

Indirect ELISA was performed to determine total anti-vimentin antibody levels in vaccinated mice and dogs. Briefly, blood samples were coagulated overnight at 4 °C and centrifuged twice at 7000 rpm for 10 min at 4 °C in a microcentrifuge. The supernatant (serum) was stored at −20 °C until use. Volumes used per well in ELISA were 50 μl, unless indicated otherwise. 96-well ELISA plates (Nunc) were coated with 4 μg/ml Vimentin protein (mouse or dog) in 0.5 M urea and then blocked with 100% horse serum (100 μl/well) (Sigma-Aldrich), both for 1 h at 37 °C. After a single wash with PBS for 1 min, the plates were incubated with serum of vaccinated animals for 45 min at 37 °C, diluted 1:10 in 100% horse serum, which was further diluted in 50% Rosetta Gami extract (final serum dilution 1:50-1:300) to reduce non-specific binding of the serum. Thereafter, plates were incubated with biotinylated polyclonal goat-anti-mouse Ig (E0433, Dako) or goat-anti-dog IgG (6070-08, Southern Biotech) for 45 min and streptavidin-HRP (P0397, Dako) for 30 min, diluted 1:2000 in 0.01% PBS-Tween-20 at 37 °C. After each incubation step, plates were washed four times with PBS. HRP activity was detected with TMB substrate (T0440, Sigma-Aldrich) and absorbance (OD) was measured at 655 nm after 15 min using a Biotek Synergy HT microplate reader (Biotek).

For specific determination of antibody titers, serial dilutions of the sera were made, and assayed as described above. Titers were calculated based on the dilution at which the OD exceeded the value of 0.2.

### RNAseq of tumors of vaccinated mice

RNA was isolated from excised B16F10 tumor tissue from TRX and TRXtr-Vimentin-vaccinated mice (*n* = 3 each), using RNeasy mini columns (Qiagen) according to the manufacturers’ recommendations. RNA was processed according to standard pipelines for expression analysis at the NKI Genomics Core Facility (Amsterdam, The Netherlands). Normalized read counts were used for further analysis using DESeq2^85^ in R studio and data were deposited in NCBI GEO database under accession number GSE172388. Gene set enrichment analysis (GSEA) was performed with GSEA 4.1.0 (https://www.gsea-msigdb.org/gsea/index.jsp) for hallmarks gene sets (h.all.v7.5.1.symbols.gmt). STRING and Enrichr were applied as described above.

### Labeling of antibodies with Zr-89

A vimentin-specific nanobody (QVQ, Utrecht, The Netherlands) was labeled with Zirconium-89 (Zr-89), to be able to determine its suitability for PET imaging, according to established procedures^86^. Briefly, the nanobodies were modified with the chelating agent NCS-Bz-Desferal by adjusting the antibody solution to pH 9.0 with Na_2_CO_3_ and reacted with 10 equivalents of NCS-Bz-Desferal for 30 min at 37˚C temperature while shaking at 550 rpm. The modified antibodies were eluted in 0.5 mL fractions containing 50 mM NaOAc/200 mM Sucrose pH 5.56. The protein concentration of the eluted fractions was determined with a NanoDrop spectrophotometer. The Desferal modified antibodies were labeled with Zr-89 at pH 6.8–7.2 in HEPES buffer for 60 min at room temperature, and showed an average of 98.0% radiochemical purity.

### PET Imaging study in B16F10 tumor-bearing mice

Exponentially growing B16F10 melanoma cells were injected subcutaneously into both flanks (2 × 10^5^/flank) of female C57BL/6 mice (*n* = 2), and grown to ~200 mm^3^.

For PET imaging, mice were anesthetized using inhalation anesthetics (isofluran 1.5 </> 2.5%; oxygen 0.45 volume %). PET images were acquired 24 h p.i. with Zr-89 labeled agent (1 MBq; retroorbital injection). During PET-CT (Mediso nanoPET-CT) mice were placed in an integrated heating bed (~35 °C) while monitoring respiratory function. Computed tomography (CT) scan was performed for ~5 min, followed by a dynamic PET scan of 60 min. PET data were normalized and corrected for scattering, randoms, attenuation, decay, and dead time. The list mode PET data were rebinned and reconstructed using an iterative 3D Poisson ordered-subsets expectation-maximization algorithm with four iterations and six subsets. The resulting images had a matrix size of 256 × 256 × 207 voxels, each with a dimension of 0.6 × 0.6 × 0.6 mm^3^ ^86^.

Immediately after the last PET scan, mice were sacrificed, blood and various tissues were excised, rinsed, dipped dry, weighed, and the amount of radioactivity determined using an LKB 1282 Compugamma CS gamma counter (LKB, Wallac). Results were expressed as a percentage of the injected dose per gram of tissue (%ID/g).

### Dog studies

Dog patients with spontaneous (recurrent) transitional cell carcinoma (TCC) of the bladder were recruited within their own veterinary practice. Upon owner consent, dogs were included in the study (approved by the local Animal Ethics Committee of the VU University and the national Central Animal Experiments Committee (CCD), AVD11400202011305) and followed regular monitoring schedules within their own veterinary practice. Dogs were vaccinated in the groin with 1 ml vaccine containing 500 µg recombinant TRXtr-Vimentin protein in 2 M urea/0.9% NaCl, 375 µg CpG 2006 oligonucleotide (5’-T*C*G*-T*C*G*-T*T*T*-T*G*T*-C*G*T*-T*T*T*-G*T*C*-G*T*T*-3’; Eurogentec) and 500 µl 10% Montanide gel 01PR (36067D, Seppic; end concentration Montanide gel 5%). Initially, dogs received four vaccinations at 2-week intervals. Where possible, tumor size was monitored by ultrasound of the bladder. Prior to inclusion in the study, an x-ray of the thorax was performed to exclude lung metastases. This interim analysis of an ongoing study includes dogs (*n* = 10) enrolled in the study between 20 February 2020 and 20 February 2021, and who had received at least three vaccinations before 1 June 2021. Four of the ten dogs had recurrent TCC for which one dog received surgery and two dogs received photodynamic therapy before inclusion in the present study. All other dogs were treated for primary disease, of which one underwent surgery and one received photodynamic therapy prior to inclusion in the current study. Adverse events and tumor responses were monitored according to VCOG criteria^37,38^.

Dogs were followed up in monthly intervals and received booster vaccinations when titers continuously dropped. All dogs received additional meloxicam, with a starting dose of 0.2 mg/kg followed by daily doses of 0.1 mg/kg. During regular visits, ultrasound and x-ray of the thorax were performed. Blood was drawn at each visit to the veterinary for determination of anti-vimentin antibody titers by ELISA, as described above.

### Image and data processing

Experiment images were acquired as detailed in the respective sections. Where necessary for visual presentation, images were globally adjusted for contrast, white balance, and/or color balance using Adobe Photoshop CS6. All quantitative data were processed in MS Excel 2010 or R Studio.

### Statistical analysis

Data are presented as means of multiple independent experiments. Error bars represent the standard error of the mean unless specified otherwise. Statistical significance was determined in GraphPad Prism^®^ version 9 using one-way analysis of variance (ANOVA) with Bonferroni multiple comparison test or using student’s *t* test, or equivalent non-parametric tests where appropriate. For analysis of tumor growth curves, two-way ANOVA was applied with Dunnett’s posthoc multiple comparison test. All tests were two-sided, and (adjusted) *p* values (if *p* < 0.05) are shown in the plots. Outliers were only excluded based on the Grubbs test (https://www.graphpad.com/quickcalcs/Grubbs1.cfm).

### Reporting summary

Further information on research design is available in the Nature Research Reporting Summary linked to this article.

## Supplementary information

Supplementary Information

Reporting Summary

## Data Availability

RNAseq data are deposited in NCBI GEO (GSE172388), and proteomics data are deposited in the PRIDE repository [https://www.ebi.ac.uk/pride/archive/projects/PXD024426]. The publicly available data used in this study are listed in Supplementary Table 8. The remaining data are available within the Article, Supplementary Information, or Source Data file. Source data are provided in this paper.

## Source data

Source Data
