# Supplementary material for: Extracellular vimentin mimics VEGF and is a target for anti-angiogenic immunotherapy
Source: Nat Commun. 2022 May 23;13:2842. doi: 10.1038/s41467-022-30063-7 (PMC9126915; doi:10.1038/s41467-022-30063-7)
Supplement: Supplementary file 1 — Supplementary Information [file 41467_2022_30063_MOESM1_ESM.pdf]

Supplementary Figure 1

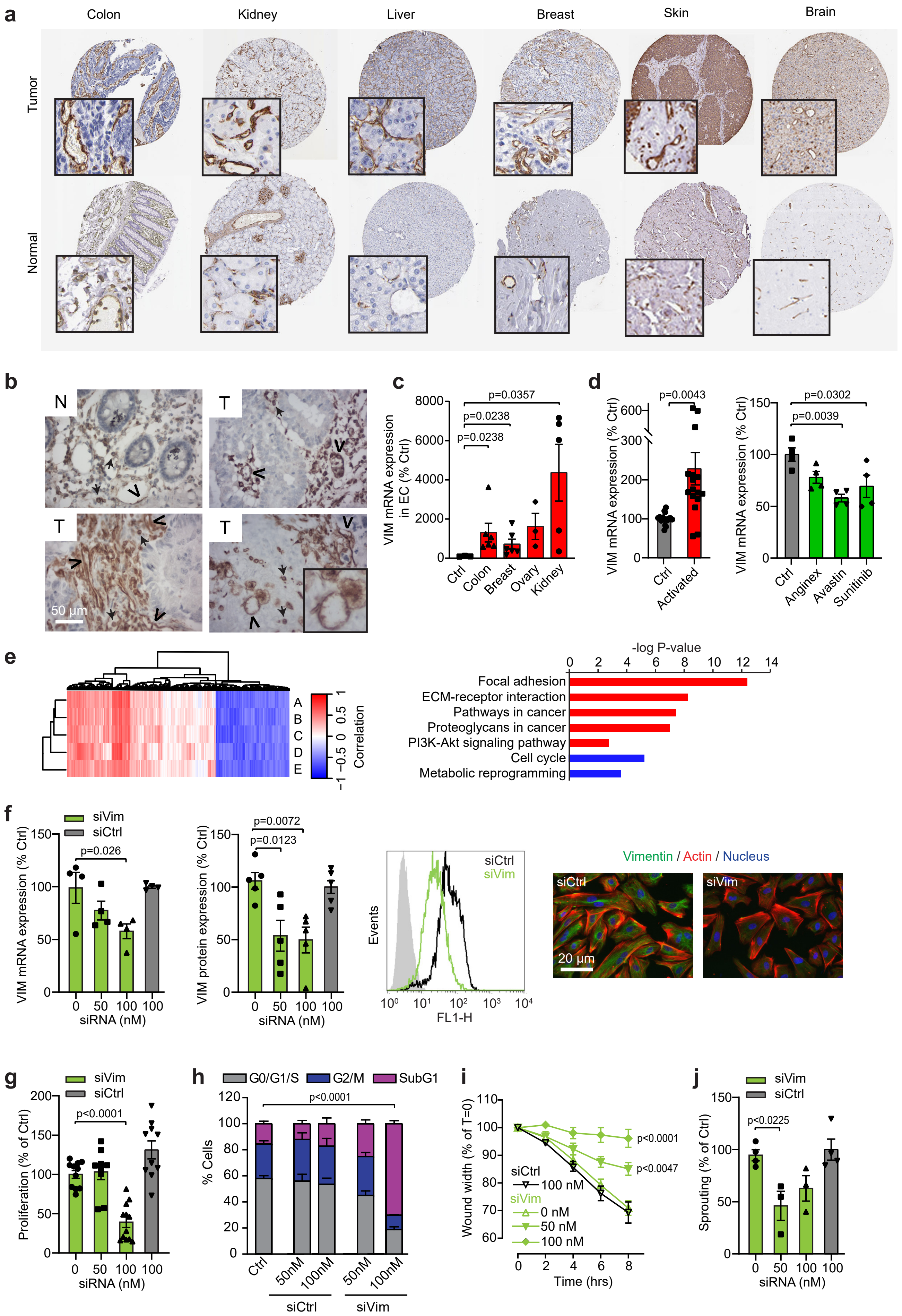

Supplementary Figure 1: Vimentin is overexpressed in tumor endothelium and associated with a pro-angiogenic phenotype

a Vimentin protein expression in tumor and normal tissue sections of colon, kidney, liver, breast, skin and brain (https://www.proteinatlas.org/). Section diameter = 1mm. Insets show 10x magnifications. Representative images are shown; number of samples per tissue are provided in the Source Data file. **b** Immunohistochemical staining of human normal colon (N) and colorectal cancer (T) for vimentin. Large arrows (V): vimentin expression in the vasculature of tumors. Small arrows: vimentin positive immune cells. Representative images are shown of n=27 CRC and n=15 normal colon samples. **c** qPCR for tumor endothelial vimentin in human tumor spheroids xenografted onto the chicken chorioallantoic membrane (CAM). Vimentin expression in host stroma is upregulated in all tumor types tested. n=3 (ctrl, ovary), n=5 (kidney), n=6 (breast, colon) eggs/tumor type. Data represent means ± SEM. p-values represent Mann Whitney test vs control. **d** qPCR of vimentin expression in untreated (Ctrl) and angiogenically activated HUVEC (left panel; n=8 different donors), as well as in HUVEC after treatment with different angiogenesis inhibitors (right panel; n=4 different donors). Data represent means ± SEM. p-values represent unpaired t-test (left panel) and one-way ANOVA with Bonferroni correction (right panel). **e** Heatmap (left) and pathway analysis (right) of (top 250) genes correlating with vimentin expression in five public gene expression data sets of colorectal cancer. Red and blue represent genes and processes positively and negatively correlated with vimentin expression, respectively. **f** Quantification of vimentin mRNA (left: qPCR, n=4) and protein (middle: flow cytometry, n=5; right: immunofluorescence staining, representative images shown) expression after transfection (48h) with siRNA targeting vimentin. Bar graphs represent means ± SEM. p-values represent one-way ANOVA with Bonferroni correction. **g-j** Effect of vimentin knockdown on HUVEC proliferation (**g**), cell cycle profiles (**h**), migration (**i**), and sprouting (**j**). All data are presented as percentage of untreated cells (Ctrl), and 0nM represents the mock transfected control, on n=4 different donors. Data represent means ± SEM. p-values represent one-way ANOVA with Bonferroni correction (**g, j**) and two-

way ANOVA with Dunnet’s correction (**h, i**). siCtrl: control non-targeting siRNA; siRNA; siVim: vimentin targeting siRNA. Source data are provided as a Source Data file.

Supplementary Figure 2

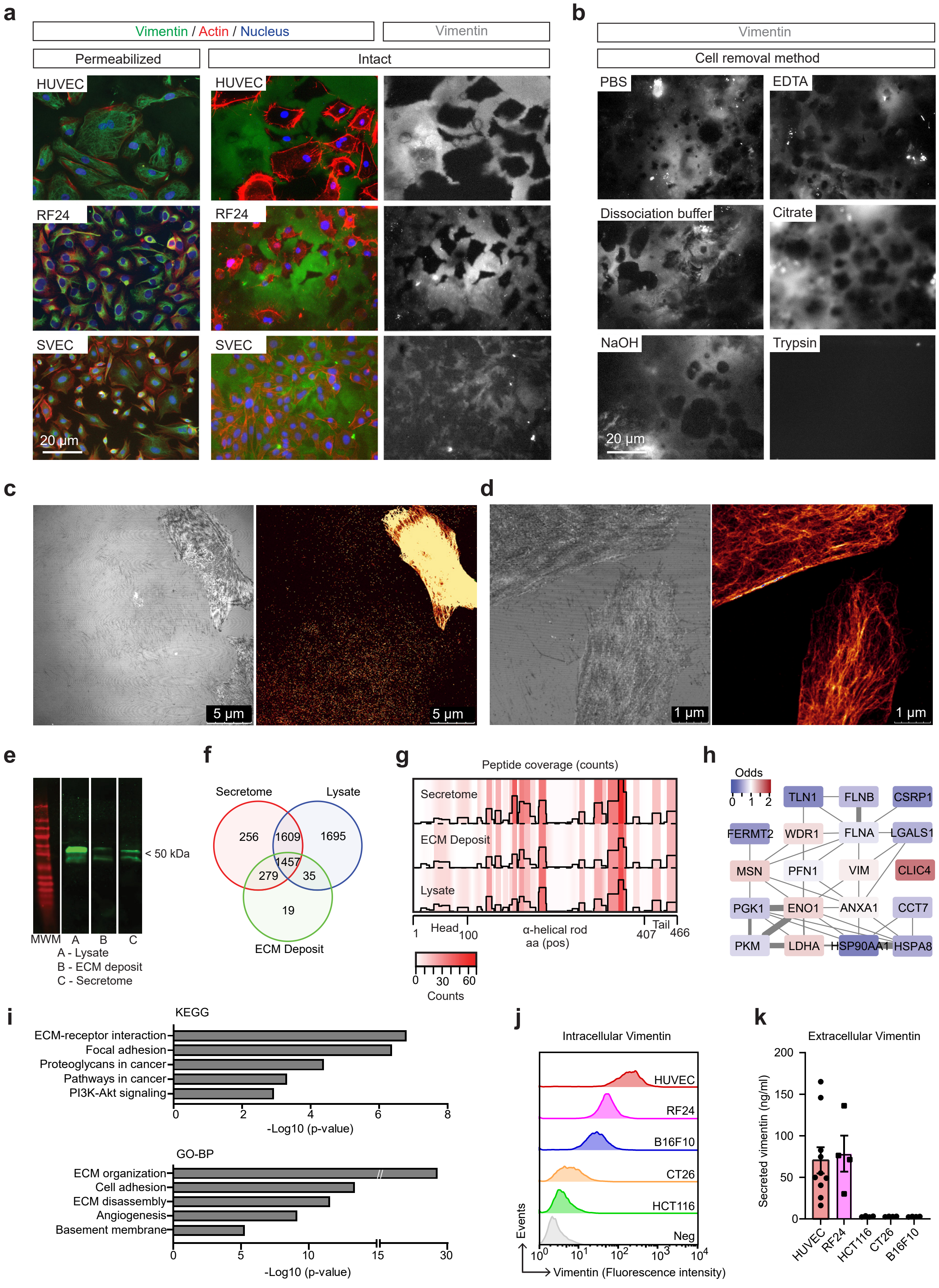

Supplementary Figure 2: Vimentin is secreted by endothelial cells and deposited in the extracellular matrix

a Immunofluorescent staining of different EC, grown on tissue culture substrates, for vimentin (left and middle panels: green; right panel: white). Cells were fixated and permeabilized (left panels) or left intact (middle and right panels), showing filamentous staining in permeabilized cells and deposited vimentin surrounding intact cells. **b** Visualizations of ECM deposits following different cell detachment methods. EC were grown on tissue culture substrates to near confluence and removed using different reagents as indicated. Non-enzymatic detachment allowed retention of vimentin deposits in the ECM. Trypsin detachment resulted in absence of staining, and confirms the proteinaceous nature of the deposits present. Representative images of at least 3 independent experiments are shown for panels **a,b**. **c, d** Differential interference contrast (left panels) and high-resolution immunofluorescence (right panels) microscopy of HUVEC. Vimentin is represented in yellow-orange. Representative images of a single experiment are shown. **e** Western blot as presented in Figure 1, with whole lanes shown. Representative sections of at least 3 independent experiments are shown. **f** Count distribution of detected proteins in HUVEC lysate, secretome and ECM deposit by global proteomics analysis. **g** Peptide coverage of vimentin in the different cellular fractions. Peptide counts per amino acid position are indicated with the black lines as well as by color intensity. No major differences in sequence coverage are seen, confirming presence of full-length protein. **h** Protein-protein interaction analysis of extracellular overexpressed proteins that have no known secretion mechanism. Colors indicate the SecretomeP odds prediction for extracellular expression. **i** Proteins significantly expressed extracellularly were subject to ontology analysis (Top: KEGG, Bottom: GO-Biological Process). Extracellular proteins are enriched for cell-cell and cell-matrix interactions and remodeling, as well as angiogenesis and cancer. **j** Intracellular vimentin protein expression of endothelial cells (EC; HUVEC, RF24) and tumor cell lines (TC; B16F10, CT26, HCT116) used in this study, as measured by flow cytometry. Representative histograms are shown. **k** Secretion of vimentin in EC and TC used in this study, as measured by

ELISA. n=10 (HUVEC) and n=4 (other). Data represent means ± SEM. Source data are provided as a Source Data file.

Supplementary Figure 3

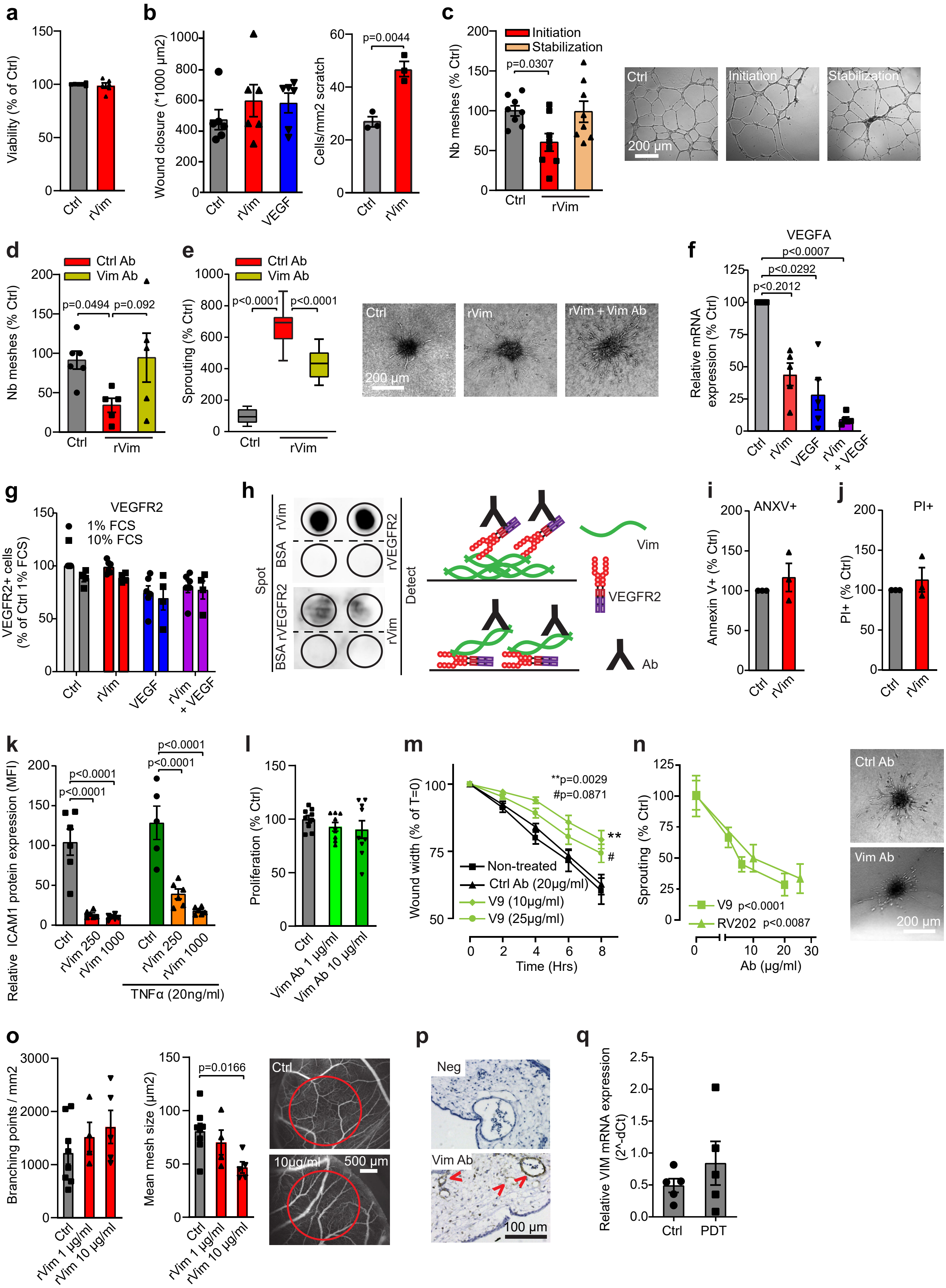

Supplementary Figure 3: Extracellular vimentin induces a pro-angiogenic endothelial phenotype

a,b Effects of rVim on EC viability (**a**) and migration (**b**; left panel). Migration of individual cells into the scratch area (**b**; right panel). n=3 different donors. p-values represent unpaired t-test. **c** Tube formation of HUVEC seeded on Matrigel directly in the presence of rVim (initiation), or after two hours (stabilization). n=4 different donors, p-values represent one-way ANOVA with Bonferroni correction. **d** HUVEC tube formation on Matrigel in the presence of rVim and/or anti-vimentin antibodies (Vim Ab). n=3 different donors. p-values represent Kruskal-Wallis test with Dunn's correction. **e** HUVEC sprouting in collagen gel in presence of rVim and/or Vim Ab. n=18 (Ctrl), n=13 (Ctrl Ab+rVim) and n=14 (Vim Ab+rVim). p-values represent one-way ANOVA with Bonferroni correction in panels. **f** VEGFA mRNA expression in HMEC-1 following treatment with rVim and/or VEGF, n=5. p-values represent Kruskal-Wallis test with Dunn's correction. **g** VEGFR2 cell surface expression in HMEC-1 following treatment with rVim and/or VEGF. n=4 (1%FCS) and n=5 (10% FCS). **h** Spotblot analysis of direct interaction between rVim and recombinant VEGFR2 or vice versa. **i, j** AnnexinV (ANXV) and propidium iodide (PI) positive Jurkat T cells after rVim treatment. n=3. **k** ICAM1 protein expression in HUVEC following treatment with rVim and/or TNFα. n=6. p-values represent one-way ANOVA with Bonferroni correction. **l** HUVEC proliferation in the presence of Vim Ab. n=3 different donors. **m, n** Migration (**m,** n=4 different donors) and sprouting (**n,** n=3 (RV202) and n=5 (V9) different donors) of HUVEC in the presence of anti-vimentin Abs p-values represent two-way ANOVA with Dunnet's correction. **o** Effects of rVim *in vivo* in the CAM. n=4 (rVim 1µg/ml), n=5 (rVim 10µg/ml), n=8 (ctrl) eggs per condition. p-values represent one-way ANOVA with Bonferroni correction. **p** Immunohistochemistry for vimentin expression in CAM. **q** mRNA expression of vimentin in control and PDT-treated CAMs. n=5. VEGF and TNFα were used at 20ng/ml. Antibodies were used at 10µg/ml. All bar graphs and X-Y plots show means ± SEM, boxplots show medians ± 10-90th percentiles. Representative images are shown, and original spot blots are presented elsewhere in the Supplementary Information. Source data are provided as a Source Data file.

Supplementary Figure 4

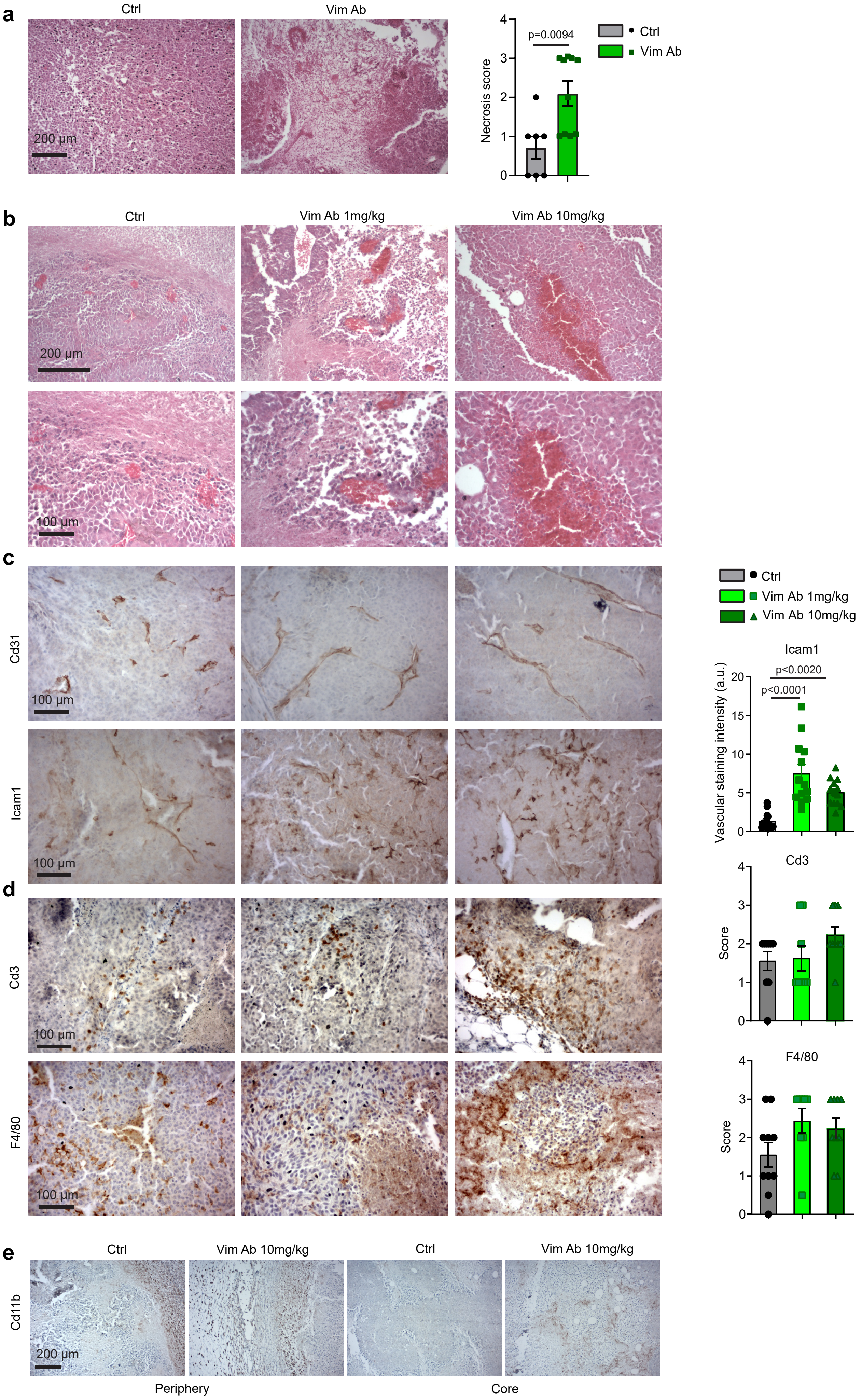

Supplementary Figure 4: Anti-angiogenic and pro-inflammatory effects of anti-vimentin antibody therapy *in vivo*

a H&E staining of human HCT116 tumors grafted on the CAM, under control treatment or anti-vimentin antibody (Vim Ab) treatment. Scoring of necrotic areas is shown in the graph. n=7 (Ctrl) and n=9 (Vim Ab) tumors per group. Data represent means ± SEM. p-values represent Mann-Whitney U test. **b** H&E staining of mouse B16F10 tumors grafted in mice, under control treatment or anti-vimentin antibody treatment. Note the reduced demarcation of blood vessels in the antibody treatment conditions, where erythrocytes are no longer bounded by intact blood vessels but rather spread in the tissue. **c** Cd31 and Icam1 staining of B16F10 tumors, under control treatment or anti-vimentin antibody treatment. Vascular Icam1 staining was quantified in ImageJ and normalized for vessel density per tissue slide (right panel). n=13 sections analyzed of n=3 tumors per condition. Data represent means ± SEM. p-values represent one-way ANOVA with Bonferroni correction. **d** Cd3 and F4/80 staining for T-cells and macrophages respectively, in B16F10 tumors, under control treatment or anti-vimentin antibody treatment. Right panels show scoring of infiltrating cells. n=10 tumors/group. Data represent means ± SEM. p-values represent Kruskal-Wallis test with Dunn's correction. **e** Cd11b expression in B16F10 tumors, under control treatment or Vim Ab treatment. Representative images are shown of n=10 tumors/group, where indicated at the periphery and core of the tumors. Source data are provided as a Source Data file.

Supplementary Figure 5

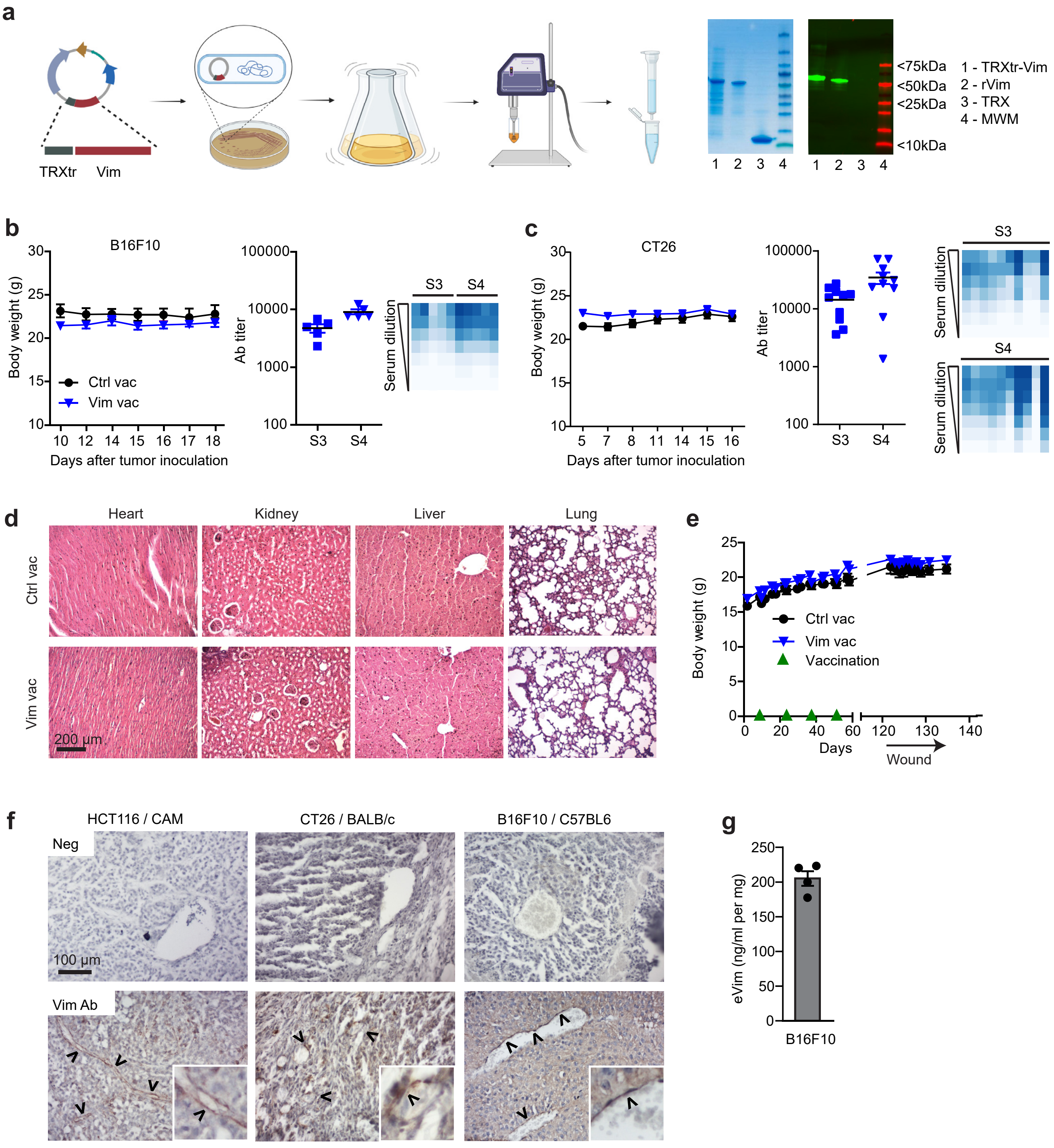

Supplementary Figure 5: Vaccine production and analysis of systemic effects of vaccination

a Schematic overview of protein expression for vaccination, created with BioRender.com. Proteins are expressed in *E.coli* and subsequently purified with Ni-Agarose. SDS-PAGE and western blot (right panels) confirm the presence of proteins of the expected molecular weight. Uncropped blots and gels are shown elsewhere in the Supporting Information. **b,c** Mice (C57BL/6 or BALB/c) were immunized with the recombinant vimentin fusion protein prior to B16F10 melanoma or CT26 colorectal carcinoma tumor cell inoculation in the flank according to the schedule presented in Figure 4. During the experiment, body weight (left panels; means ± SEM) and individual antibody titers (right panels) were monitored. Heatmaps of OD655nm ELISA values on serum dilutions of the individual vimentin vaccinated mice, used to calculate the anti-vimentin antibody titers, are shown on the side. n=5 (B16F10, **b**) and n=10 (CT26, **c**) mice/group. **d** H&E staining of different organs from mice (C57BL/6, n=5) that received control or vimentin vaccination, and remained hyperimmune for 40 weeks. Representative images are shown. **e** Body weight of control and vimentin vaccinated mice during the wound healing study. Data represent means ± SEM, n=5 mice/group. **f** Immunohistochemical staining of tumors from models used in this study. Top panels show negative control stainings whereas bottom panels show staining for vimentin. Insets highlight vimentin staining present predominantly in the vasculature (arrows). Representative images are shown of n=9 (HCT116 / CAM), n=10 (CT26 / Balb/c) and n=5 (B16F10 / C57BL/6) tumors.. **g** Secreted vimentin detected in the secretome of B16F10 tumors in mice by ELISA. Data represent means ± SEM, n=4. Source data are provided as a Source Data file.

Supplementary Figure 6

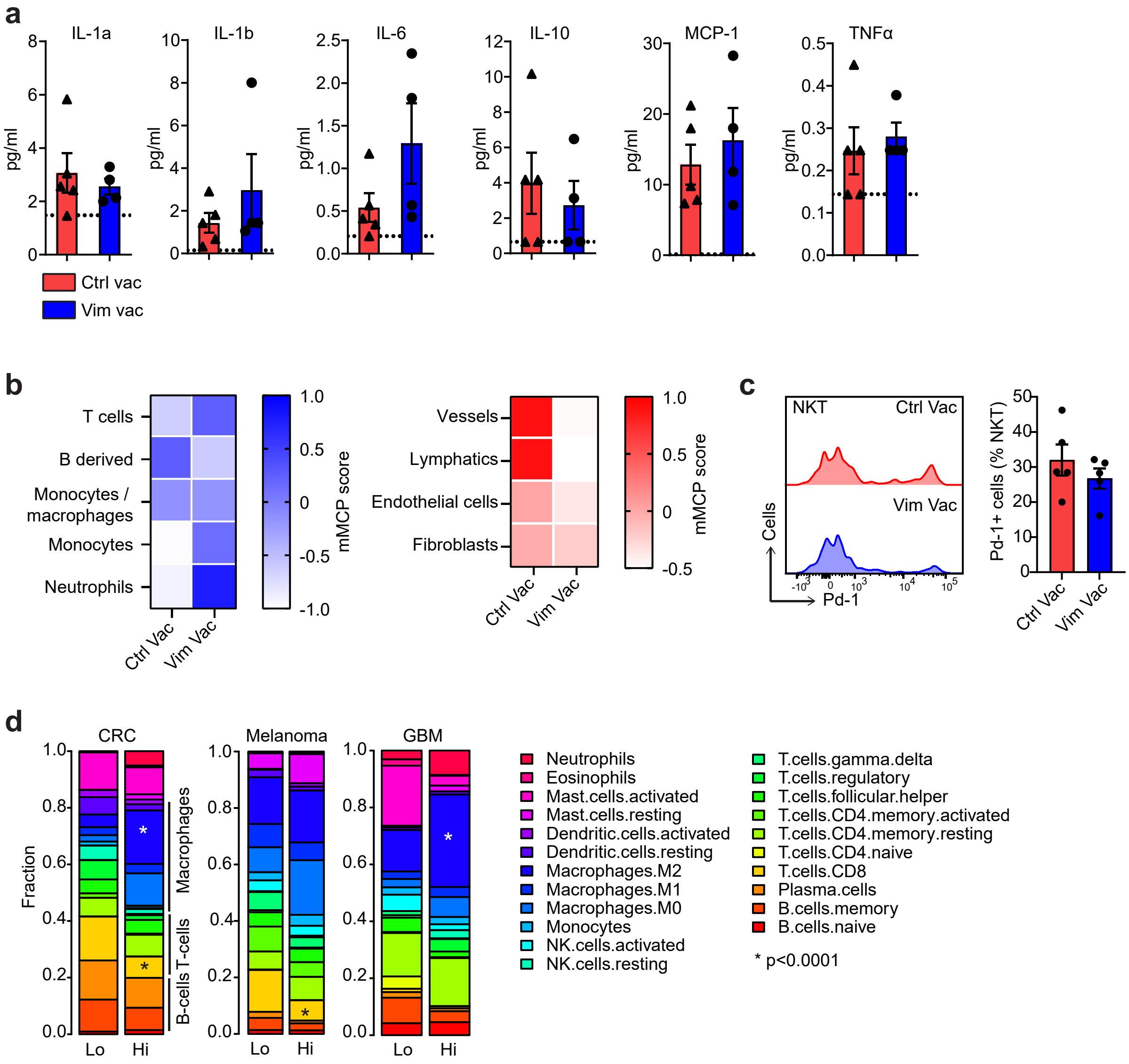

Supplementary Figure 6: Immunomodulatory effects of vimentin vaccination and expression

a Cytokine profiling in secretome of B16F10 tumors of vimentin and control vaccinated mice, using bead-based flowcytometry. Dotted lines indicate the detection threshold. Data represent means ± SEM, n=4 (Vim Vac) and n=5 (Ctrl Vac). **b** *In silico* analysis using the mMCP-counter method on RNAseq data of B16F10 tumors of vimentin and control vaccinated mice. The left panel summarizes the relative presence of immune cell subsets whereas the right panel summarizes the relative presence of stromal cell subsets. **c** Pd-1 expression on NKT cells detected in B16F10 tumors of vimentin and control vaccinated mice by flow cytometry. Histograms represent concatenated data of n=5 mice/group. Bar graphs represent means ± SEM of n=5 mice/group. **d** Digital flow cytometry using Cibersort of immune cell infiltrate subsets human CRC (GSE17538, n=24), melanoma (GSE65904, n=21) and glioma (GSE4290, n=10), stratified for the 10% highest and 10% lowest vimentin expressing tumors in the given data set. p-values represent two-way ANOVA with Bonferroni correction. Source data are provided as a Source Data file.

Supplementary Figure 7

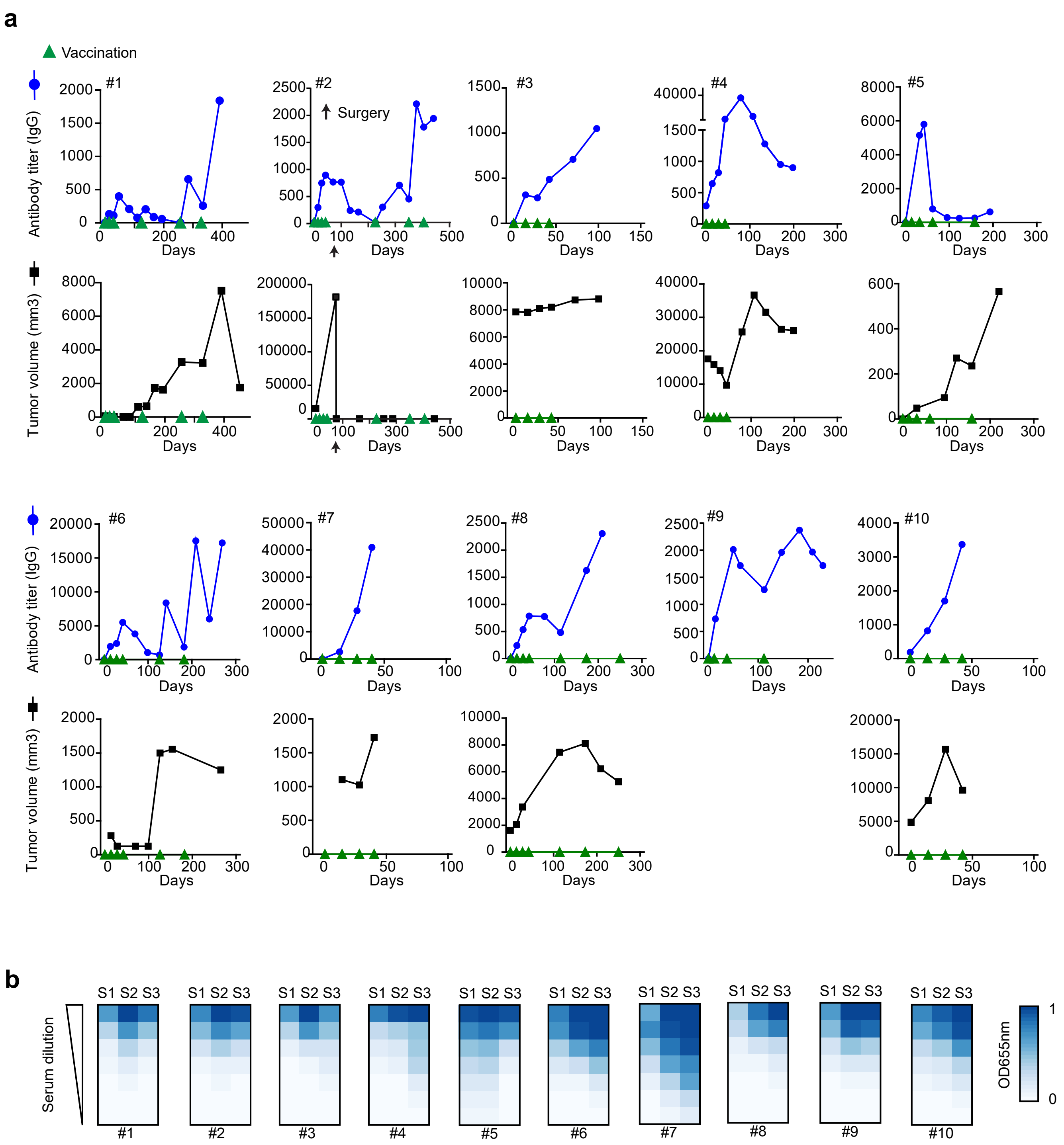

Supplementary Figure 7: Antibody titers and tumor volumes after Vimentin vaccination in dogs

a Titers (blue), vaccinations (green) and tumor size estimates (black) for individual dogs in the study. Note that for dog #9 no ultrasound data (tumor size) was available. **b** Heatmap representation of OD655nm values in ELISA on serially diluted serum for determination of antibody titers after the first three vaccinations in all dogs. Source data are provided as a Source Data file.

Supplementary Figure 8

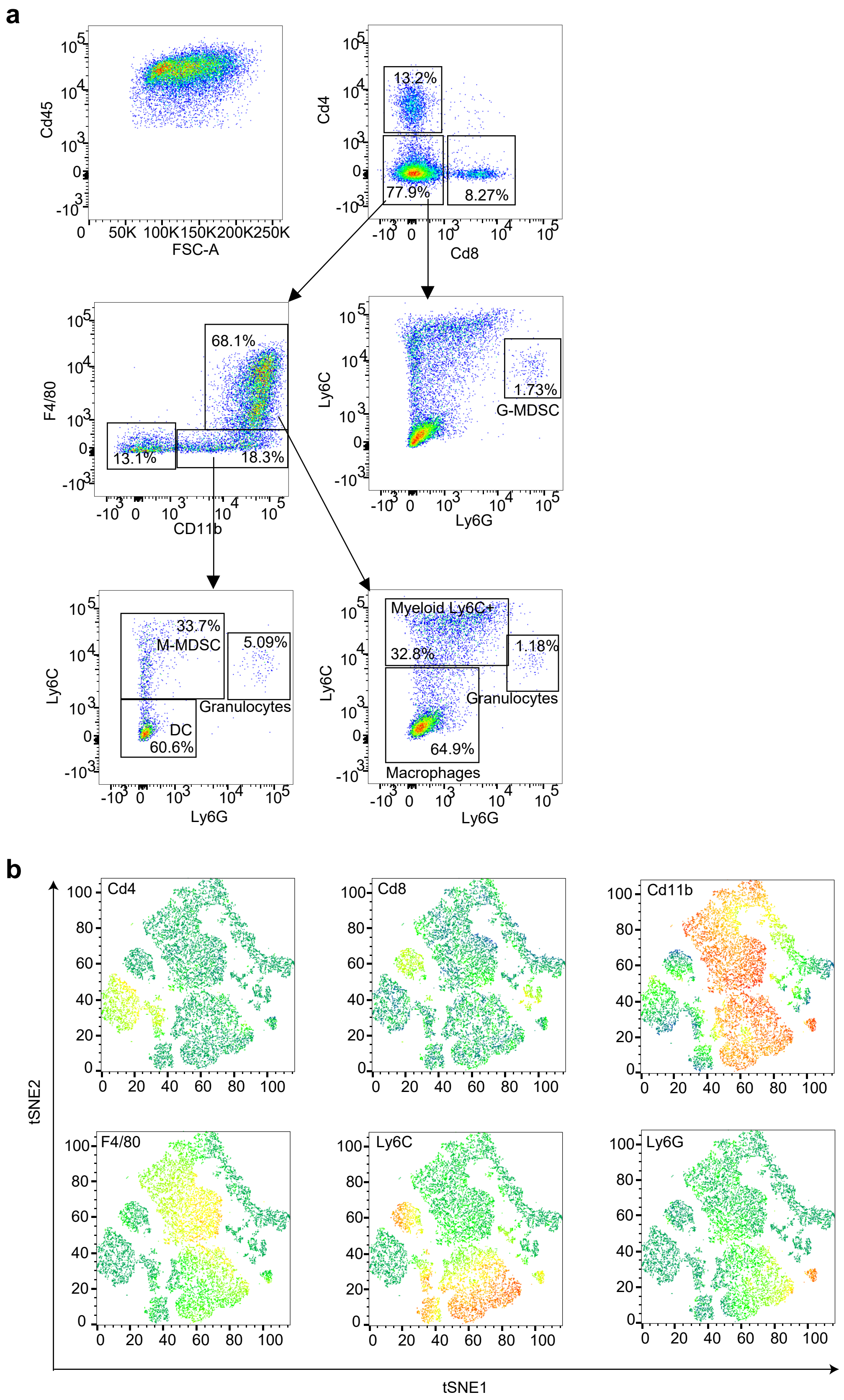

Supplementary Figure 8: Flow cytometry gating (I)

a Gating strategy associated with Figure 5i. Cell populations were pregated on live, single-cells.

b Marker expression visualized in tSNE plots.

Supplementary Figure 9

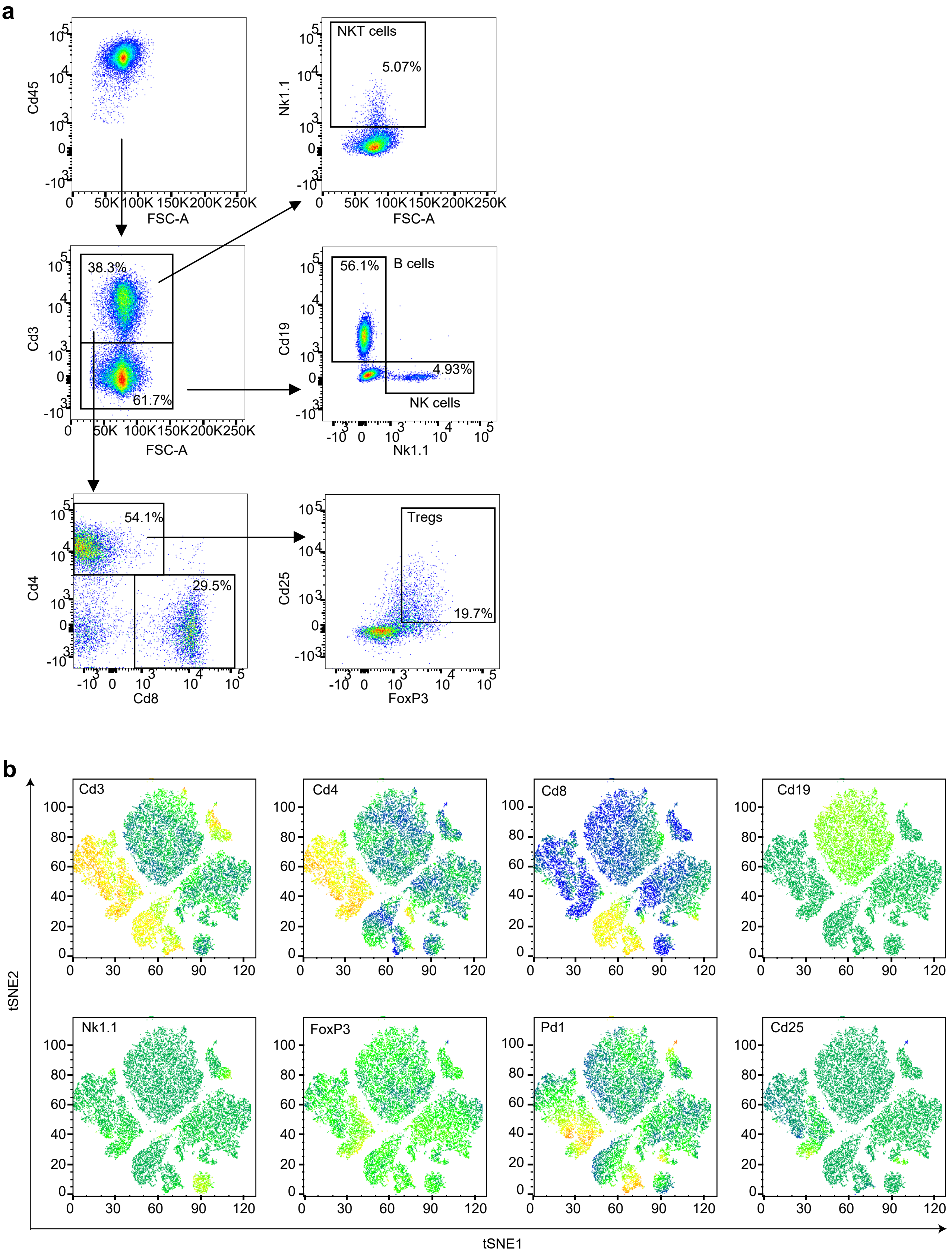

Supplementary Figure 9: Flow cytometry gating (II)

a Gating strategy associated with Figure 5j. Cell populations were pregated on live, single-cells.

b Marker expression visualized in tSNE plots.

Supplementary Figure 10

a

Original blot - Figure 1g, Supplementary Figure 2e

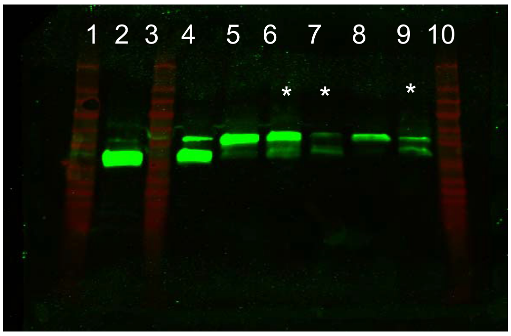

Lane
1 Molecular weight marker (MWM)
2 Trypsin cells 75ul
3 MWM
4 Trypsin cells 25ul
5 Cells citrate
6 *Total scrape
7 *Deposit trypsin
8 Deposit citrate
9 *CM
10 MWM
* Presented lanes Fig 1g, Sup 2e

b

Original blots - Supplementary Figure 3h

(i) rVim on spotted rVEGFR2

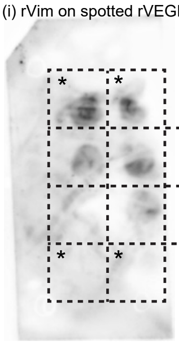

rVEGFR2 - A
hVim
V9
rVEGFR2 - B
hVim
V9
rVEGFR2 - B
hVim
V9
BSA
hVim
V9

(ii) rVEGFR2 on spotted rVim (iii) rVEGFR2 on spotted BSA

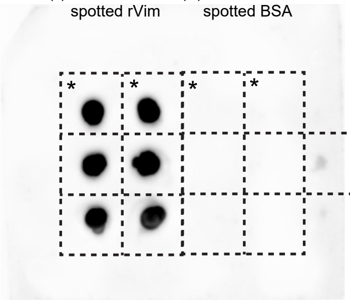

(ii)	(iii)
hVim<br>rVEGFR2 - A<br>VEGFR2 Ab	BSA<br>rVEGFR2 - A<br>VEGFR2 Ab
hVim<br>VEGFR2 - B<br>VEGFR2 Ab	BSA<br>VEGFR2 - B<br>VEGFR2 Ab
hVim<br>VEGFR2 - C<br>VEGFR2 Ab	BSA<br>VEGFR2 - C<br>VEGFR2 Ab

Spotted protein
Interacting protein
Detector Ab

c

Original blots - Supplementary Figure 5a

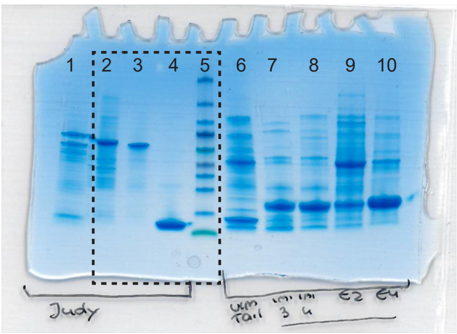

Lanes in figure
2 TRXtr-Vim
3 hVim
4 TRXtr
5 MWM

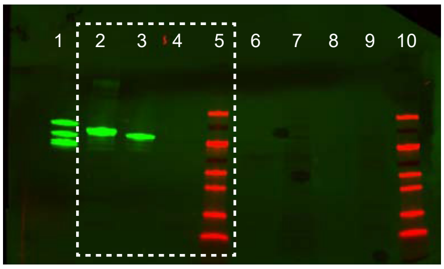

Lanes in figure
2 TRXtr-Vim
3 hVim
4 TRXtr
5 MWM

Supplementary Figure 10: Original gels and blots

a Original blot of representations in Figure 1g and Supplementary Figure 2e. Lanes indicated with an * are shown in the figure panels. **b** Original spotblots of representations in Supplementary Figure 3h. Boxes indicated with an * are shown in the referred figure panel. The dots are separated by dashed lines, with to the right the order of incubation. A general setup is shown in italics on the side referring to the order of spotting, protein incubation, and antibody detection as illustrated in Supplementary Figure 3h. **c** Original gel and blot of representations in Supplementary Figure 5a. Boxes are shown in the referred figure panel.

Supplementary Table 1 - Compounds

Compound	Concentration Range	Mechanism / function	Supplier	Code
**Growth factors & Cytokines**				
bFGF	1-100ng/ml	Angiogenic factor	Preprotech	100-18B
VEGF	1-100ng/ml	Angiogenic factor	Preprotech	100-20
TGFb	1-100ng/ml	Growth factor / cytokine	R&D systems	240-B
TNFa	1-100ng/ml	Proinflammatory cytokine	BioLegend	57010x
IFNy	50-200U/ml	Proinflammatory cytokine	BioLegend	57020x
IL-10	1-100ng/ml	Cytokine; inhibits PKC, blocks secretion; 10-500ng/ml by Mor-Vaknin	BioLegend	57100x
**TKI/anti-angiogenic drugs**				
Sunitinib	0.1-10uM	VEGFR TKI	Pfizer	PF-00262192-41
Avastin	1-100ug/ml	Anti-VEGF Ab	Roche	B8507H28
AZD4547	0.5-50uM	FGFR inhibitor	Selleckchem	S2801
Dasatinib	0.001-20uM	BCR/Abl inhibitor	LClabs	D-3307
Crenolanib	0.5-50uM	PDGFR inhibitor	Selleckchem	S2730
Sorafenib	0.1-10uM	VEGFR TKI	Bayer	BAY-43-9006
**Glycosylation**				
Heparin	1-100ug/ml	Release of BM bound GF, interaction with ECM	Sigma	H0200000
Hyaluronidase	1-100ug/ml	Degradation of hyaluronan	Sigma	H3884
Tunicamycin	0.1-10ug/ml	Inhibition of N-acetylglucosamine transferases; inhibition of glycosylation of proteins; induction of ER stress	Sigma	T7765
**Membrane function**				
Digoxin	0.1uM-100uM	Na+/K+ pump inhibitor; cardenolide; affects FGF2 release	Sigma	D6003
Glyburide (Glibenclamide)	1uM-100uM	Inhibits ABC1 transporter activity; affects IL1b secretion	Santa Cruz	sc-200982A
N-Ethylmaleimide (NEM)	10nM-10mM	Used as inhibitor of flippases	Sigma	E3876
Ouabain	10uM-100uM	Na+/K+ pump inhibitor; cardenolide; affects FGF2 release	Sigma	O3125
Verapamil	0.1-100uM	Calcium channel inhibitor/ Ca2+ionophore/PgP inhibitor	Sigma	381195
**Protein trafficking**				
3-methyladenine (3-MA)	1uM-1mM	PI3K inhibitor; autophagosome inhibitor	Sigma	M9281
Bafilomycin A1	1nM-10uM	Prevents maturation of autophagic vacuoles by inhibiting fusion between autophagosomes and lysosomes	LClabs	B-1080
Brefeldin A	0.5-10ug/ml	Protein transport blocker; ER & Golgi	Life Technologies	00-4506-51
Carbonyl cyanide chlorophenylhydrazone (CCCP)	0.1 -10uM	Uncoupling oxidative phosphorylation;Disruption of lysosomal pH	Sigma	C2759
D-Sphingosine	1-100uM	PKA inhibitor	Sigma	S6879
Exo1	10-10uM	Exocytose inhibitor	Sigma	E8280
Forskolin	1-50uM	cAMP inducer; FABP4 release induction from adipocytes	ICM Biomedicals	199669
GW4869	0.1-5uM	Exosome inhibitor; nSmase-2 inhibition	Sigma	D1692
Histamine	20-100uM	Promotion of MVB-PM fusion and the release of CD63-enriched exosomes	Sigma	H7250
Leupeptin	10-100uM	Lysosome inhibitors; inhibition of a-synuclein secretion	Sigma	L9783
Methylamine	1 100mM	Inhibitor of endocytosis/endosomal recycling; Inhibition of autophagy; endo/exocytosis	Sigma	M0505
Monensin	0.5- 25nM	Protein transport blocker; Vesicles & Golgi	Sigma	M5273
Neomycin	1-10mM	Blocking protein interactions with phosphoinositides	Sigma	N6386
NH4Cl	10-100mM	Disruption of lysosomal pH	JT Baker	0019
Nigericin	2 -20uM	NLRP3 inlammasome agonist; autophagy support	Sigma	N7143
Nocodazole	10-100uM	Interference microtubules; autophagy	Sigma	M1404
Pepstatin A	0.5-1ug/ml	Lysosome inhibitors; inhibition of a-synuclein secretion	Sigma	P4265
Rapamycin	1nM-10uM	Inducer of autophagy; Anti-proliferative; mTOR1 inhibitor	LClabs	R-5000
Thrombin	1U/ml	Activation of secretion of vWF from EC. Affects secretion FGF2 and PDI secretion from HUVEC	Sigma	T7513
Tubacin	0.1-10uM	HDAC6 inhibitor; HSP90 inhibitor - HDAC deacetylates HSP90	Selleckchem	S2239

Supplementary Table 2 - Dogs

Breed	Age (y)	Sex [a]	Weight (kg)	Prim/Rec [b]	Location	PreviousTX	Response [c]	Survival (d)	AE (grade1-5)	Study status	Remarks
Galgo español	11.9	FS	26.6	R	Fundus	Surgery	CR	466	2	active	
Bernese mountain dog	7.0	MC	68	P	Fundus	N	CR	454	1	active	
Podenco ibicenco	13.9	MC	19	R	Fundus	PDT	SD	108	1	died	Euthanized, due to progressive local TCC
German hunting terrrier	11.1	MC	23.9	R	Apex	PDT	SD	224	1	died	Euthanized, due to unrelated GI poblems
Boomer	9.9	FS	6.15	P	Trigone	Surgery	SD	361	2	inactive	Withdrawn after initial series of 3 vaccinations
Small dutch waterfowl dog	8.9	FI	11.75	P	Diffuse	N	SD	280	1	active	
Yorkshire terrier	10.3	FS	4.4	R	Neck	N	PR	124	1	died	Euthanized, days after operation for progressive TCC
Lagotto romagnolo	14.2	FS	13	P	Fundus	N	SD	263	1	active	
Labrador retriever	11.8	FS	31.6	P	Urethra	PDT	SD	204	1	active	
Australian shepherd	7.2	MC	26.5	P	Apex	N	SD	103	1	active	

a - FS: Female Spayed;FI: Female Intact; MC: Male Castrated

b - Prim: Primary; Rec: Recurrent

c - CR: complete remission; PR: partial remission; SD: stable disease

Supplementary Table 3 - Kits and specific reagents

Reagent	Supplier	Cat. No
Recombinant Vimentin	SinoBiologicals	10028-H08B
Recombinant Human TNF-α	Preprotech	300-01A
Recombinant Human VEGF165	Preprotech	100-20
Recombinant VEGFR2-Fc	BioLegend	595004
Human Phospho-VEGFR2/KDR DuoSet IC ELISA	R&D Systems	DYC1766-2
LEGENDplex Mouse inflammation panel	BioLegend	74046
CellTiter-Glo®	Promega	G7571
siRNA - Vimentin	Eurogentec	SR-CL002-005
siRNA - Ctrl	Eurogentec	SR-CL000-005
HiPerfect transfection reagens	Qiagen	301705
RNeasy Mini Kit	Qiagen	74104
Type I bovine collagen	Advanced BioMatrix	5005-100ML
Collagenase IV	Sigma	C5138
DNAse I type II	Sigma	D4527
Hyaluronidase type V	Sigma	H6254
Micro BCA™ Protein Assay Kit	ThermoFisher Scientific	23235
iQ SYBR™ Green Supermix	Bio-Rad	1708886
iScript™ cDNA Synthesis Kit	Bio-Rad	1708890
ECL Pico Plus reagent	ThermoFisher Scientific	34579
Biotin Protein Labeling kit	Roche	11 418 165 001

Supplementary Table 4 - Antibodies

A. Protein detection assays

Figure	Technique	Primary - Ag / Clone	Species	Cat No	Supplier	Dilution	Secondary detection	Cat No	Supplier	Dilution	Tertiary detection	Cat No	Supplier	Dilution
1B	Flowcytometry	Vimentin	Rabbit	Ab71144	Abcam	1:100	swine anti-rabbit FITC	F0205	DAKO	1:200				
1B	Flowcytometry	CD31-PE	Mouse	MCA1738PE	Serotec	1:50								
1E	Immunofluorescence	V9	Mouse	M0725	DAKO	1:100	goat anti-mouse biotin	E0433	DAKO	1:200	Streptavidin-A488	S11223	Life Technologies	1:500
1G	Western blot	E-5	Mouse	sc373717	Santa Cruz	1:1000	goat anti-mouse IRDye 800	LI 926-32210	LiCor	1:10000				
1K, M	ELISA	V9	Mouse	M0725	DAKO	1:750	goat anti-mouse biotin	E0433	DAKO	1:1500	Streptavidin_HRP	P0397	DAKO	1:3000
2C	Immunofluorescence	VE-cadherin	Rabbit	160840	Cayman	1:100	swine anti-rabbit biotin	E0431	DAKO	1:200	Streptavidin-A488	S11223	Life Technologies	1:500
2H	ELISA	Human Fc	Goat; biotin	673171	MP Biomedicals	1:1500	Streptavidin_HRP	P0397	DAKO	1:3000				
4E, F, G	ELISA	Mouse sera					goat anti-mouse biotin	E0433	DAKO	1:2000	Streptavidin_HRP	P0397	DAKO	1:2000
6B,C,E	ELISA	Dog sera					goat anti-dog biotin	6070-08	Southern Biotech	1:2000	Streptavidin_HRP	P0397	DAKO	1:2000

Supplementary Figure	Technique	Primary - Ag / Clone	Species	Cat No	Supplier	Dilution	Secondary detection	Cat No	Supplier	Dilution	Tertiary detection	Cat No	Supplier	Dilution
S1F	Flowcytometry	V9	Mouse	M0725	DAKO	1:100	goat anti-mouse FITC	F0479	DAKO	1:200				
S1F	Immunofluorescence	V9	Mouse	M0725	DAKO	1:100	goat anti-mouse biotin	E0433	DAKO	1:200	Streptavidin-A488	S11223	Life Technologies	1:500
S2A	Immunofluorescence	E-5	Mouse	sc373717	Santa Cruz	1:100	goat anti-mouse biotin	E0433	DAKO	1:200	Streptavidin-A488	S11223	Life Technologies	1:500
S2B, C, D	Immunofluorescence	V9	Mouse	M0725	DAKO	1:100	goat anti-mouse biotin	E0433	DAKO	1:200	Streptavidin-A488	S11223	Life Technologies	1:500
S2E	Western blot	E-5	Mouse	sc373717	Santa Cruz	1:1000	goat anti-mouse IRDye 800	LI 926-32210	LiCor	1:10000				
S2J	Flowcytometry	E-5	Mouse	sc373717	Santa Cruz	1:200	goat anti-mouse FITC	F0479	DAKO					
S2K	ELISA	E-5	Mouse	sc373717	Santa Cruz	1:750	goat anti-mouse biotin	E0433	DAKO	1:1500	Streptavidin_HRP	P0397	DAKO	1:3000
S3G	Flowcytometry	VEGFR2	Goat	AF357	R&D Systems	1:50	rabbit anti-goat biotin	E0466	DAKO	1:200	Streptavidin-A488	S11223	Life Technologies	1:500
S3H	Spotblot	V9	Mouse	M0725	DAKO	1:100	goat anti-mouse biotin	E0433	DAKO	1:500	Streptavidin_HRP	P0397	DAKO	1:1000
S3H	Spotblot	VEGFR2	Mouse	dyc1766	R&D Systems	1:100	goat anti-mouse biotin	E0433	DAKO	1:500	Streptavidin_HRP	P0397	DAKO	1:1000
S3K	Flowcytometry	ICAM1	Mouse	MON1132	Monosan		goat anti-mouse FITC	F0479	DAKO	1:200				
S5A	Western blot	E-5	Mouse	sc373717	Santa Cruz	1:1000	goat anti-mouse IRDye 800	LI 926-32210	LiCor	1:10000				
S5B, C	ELISA	Mouse sera					goat anti-mouse biotin	E0433	DAKO	1:2000	Streptavidin_HRP	P0397	DAKO	1:2000
S5G	ELISA	E-5	Mouse	sc373717	Santa Cruz	1:750	goat anti-mouse biotin	E0433	DAKO	1:1500	Streptavidin_HRP	P0397	DAKO	1:3000
S7A,B	ELISA	Dog sera					goat anti-dog biotin	6070-08	Southern Biotech	1:2000	Streptavidin_HRP	P0397	DAKO	1:2000

B. In vitro and in vivo assays

Figure	Technique	Clone	Species	Cat No	Supplier	Ag
3A	In vitro migration	V9	Mouse	M0725	DAKO	Vimentin
3A	In vitro migration	9E10	Mouse	1667 203	Roche	Ctrl antibody; myc
3B,C	In vitro tube formation	E-5	Mouse	sc373717	Santa Cruz	Vimentin
3B,C	In vitro tube formation	9E10	Mouse	1667 203	Roche	Ctrl antibody; myc
3D, E, F	In vivo CAM	RV202	Mouse	sc32322	Santa Cruz	Vimentin
3D, E	In vivo CAM	9E10	Mouse	1667 203	Roche	Ctrl antibody; myc
3G,J	In vivo CAM and mouse xenograft	TE6	Mouse	IgM	In-house developed	Vimentin
3L	In vivo immunoPET	Nanobody	Camelid	Q60	QVQ	Vimentin

Supplementary Figure	Technique	Clone	Species	Cat No	Supplier	Ag
S3D	In vitro tube formation	E-5	Mouse	sc373717	Santa Cruz	Vimentin
S3D	In vitro tube formation	HP6062	Goat	9220-01	SouthernBiotech	IgG Kappa
S3E	In vitro sprouting	RV202	Mouse	sc32322	Santa Cruz	Vimentin
S3E	In vitro sprouting	HP6062	Goat	9220-01	SouthernBiotech	IgG Kappa
S3L, M	In vitro proliferation, migration	V9	Mouse	M07525	DAKO	Vimentin
S3N	In vitro sprouting	V9	Mouse	M07525	DAKO	Vimentin
S3N	In vitro sprouting	RV202	Mouse	sc32322	Santa Cruz	Vimentin

Supplementary Table 5 - IHC Antibodies and specifications

Tissue	Fixation	Primary Ab	Clone	Species	Cat No	Supplier	Dilution	Secondary Ab	Details	Dilution	Detection	Details	Dilution	H2O2 Tx	Ag retrieval / postfixation		Block
CAM	FFPE	Vim	RV202	Mouse	sc32322	Santa Cruz	1:100	Goat-anti-mouse biotin	E0433 DAKO	1:500	Streptavidin_HRP	P0397 DAKO	1:200	1% / PBS	10mM NaCitrate pH6	Microwave	3% BSA
	FFPE	CD31	SZ31	Rat	DIA310M	Diaova	1:50	Donkey ant-rat biotin	712-067-003 Jackson	1:500	Streptavidin_HRP	P0397 DAKO	1:200	0.3% / PBS	10mM NaCitrate pH6	Microwave	3% BSA
Human	FFPE	Vim	E-5	Mouse	sc373717	Santa Cruz	1:2000	Goat-anti-mouse biotin	E0433 DAKO	1:500	Streptavidin_HRP	P0397 DAKO	1:200	1% / PBS	10mM NaCitrate pH6	Microwave	3% BSA
Mouse	FFPE	Vim	E-5	Mouse	sc373717	Santa Cruz	1:100	Goat-anti-mouse biotin	E0433 DAKO	1:500	Streptavidin_HRP	P0397 DAKO	1:200	1% / PBS	10mM NaCitrate pH6	Microwave	3% BSA
	FFPE	Vim	RV202	Mouse	sc32322	Santa Cruz	1:100	Goat-anti-mouse biotin	E0433 DAKO	1:500	Streptavidin_HRP	P0397 DAKO	1:200	1% / PBS	10mM NaCitrate pH6	Autoclave	3% BSA
	FFPE	Cd31	SZ31	Rat	DIA310M	Diaova	1:50	Donkey ant-rat biotin	712-067-003 Jackson	1:500	Streptavidin_HRP	P0397 DAKO	1:200	0.3% / PBS	10mM NaCitrate pH6	Microwave	3% BSA
	FFPE	F4/80	Cl:A3-1	Rat	MCA497A	Serotec	1:400	Donkey ant-rat biotin	A1100-137B Bethyl Laboratories	1:200	Streptavidin_HRP	P0397 DAKO	1:200	3% /MetOH	proteinase-K (P6556, 20µg/ml, Sigma-Aldrich)	37C 20'	4% BSA/5% normal goat serum
	FFPE	Pd-l1	10F.9G2	Rat	124302	Biologend	1:100	Donkey ant-rat biotin	712-067-003 Jackson	1:200	Streptavidin_HRP	P0397 DAKO	1:200	3% /PBS	proteinase-K (P6556, 20µg/ml, Sigma-Aldrich)	37C 20'	4% BSA/5% normal goat serum
	FFPE	Icam1		Goat	AF796	R&D Systems	1:100	Rabbit anti-goat biotin	E0466 Dako	1:500	Streptavidin_HRP	P0397 DAKO	1:200	3% /PBS	10mM NaCitrate pH6	Autoclave	3% BSA/PBS
	FFPE	Cd11b		Rabbit	Ab133357	Abcam	1:4000	Swine anti-rabbit biotin	E0353 DAKO	1:500	Streptavidin_HRP	P0397 DAKO	1:200	0.3% / PBS	10mM NaCitrate pH6	Autoclave	3% BSA/PBS
	FFPE	Cd3	SP7	Rat	RM-9107-S	Thermo fisher	1:600	Donkey ant-rat biotin	A1100-137B Bethyl Laboratories	1:200	Streptavidin_HRP	P0397 DAKO	1:200	3% /MetOH	Tris/EDTA pH 9.0	Autoclave	4% BSA/5% normal goat serum

Supplementary Table 6 - Flow cytometry antibody panels

Myeloid panel - Fig 5I

Antigen	Clone	Species	Cat No	Supplier	Conjugate	Dilution
F4/80	BM8	Rat	123123	BioLegend	Pacific Blue	1:20
Ly6C	HK1.4	Rat	128033	BioLegend	BV510	1:20
Ly6G	RB6-8C5	Rat	11-5931-82	eBioscience/ Thermo Fisher	FITC	1:20
Cd4	GK1.5	Rat	12-0041	eBioscience/ Thermo Fisher	PE	1:20
Cd11b	M1/70	Rat	552850	BD bioscience	PE-Cy7	1:20
Cd45	30-F11	Rat	103112	BioLegend	APC	1:20
Cd8a	53-6.7	Rat	100713	BioLegend	APC-Cy7	1:20

Lymphoid panel - Fig 5J

Antigen	Clone	Species	Product code	Supplier/source	Conjugate	Dilution
Cd45	30-F11	Rat	103128	BioLegend	AF700	1:20
Cd3	17A2	Rat	100219	BioLegend	PE-Cy7	1:20
Cd8a	53-6.7	Rat	100765	BioLegend	APC-Fire750	1:20
Cd4	RM4-5	Rat	100552	BioLegend	BV785	1:20
CD19	6D5	Rat	115524	BioLegend	AF488	1:20
Nk1.1	PK136	Mouse	108753	BioLegend	BV605	1:20
Cd25	PC61	Rat	102043	BioLegend	BV421	1:20
FoxP3	MF-14	Rat	126407	BioLegend	AF647	1:20
Ctla-4	UC10-4B9	Hamster	106305	BioLegend	PE	1:20
Pd-1	29F.1A12	Rat	135231	BioLegend	BV711	1:20

Supplementary Table 7 - qPCR primers

CAM tumors	Fw	Rev
hs_VIM	GCGAGGAGAGCAGGATTTCTC	ACCAGAGGGAGTGAATCCAGA
gg_VIM	GGAGAAGAGAGCAGGATTAACA	TCAACAATTGGCTGAGACTCA
hs_ACTB	TTCCTATGTGGGCGACGAG	TCCTCGGGAGCCACACG
gg_ACTB	AGACAGCTACGTTGGTGATGAA	TGCTCCTCAGGGGCTACTCT
hs_PPIA	AGCATGTGGTGTTTGGCAAA	TCGAGTTGTCCACAGTCAGC
gg_PPIA	AAGGAGGGGATGAACGTG	AGCTGCCCGCAGTTGGA
hs_B2M	TCCATCCGACATTGAAGTTG	CGGCAGGCATACTCATCTT
gg_B2M	AGGATCACCATCACGCTGA	TGTAGACGGCTTCGCTGC

Mouse tumors	Fw	Rev
mm_Vim	CACACTTGGTGCAACAGTGC	GGGTGTCAGTTGTTAAGTGCTG
mm_Icam1	GTGGCGGGAAAGTTCCTG	CGTCTGCAGGTCATCTTAGGAG
mm_Pd-l1	CGTGAGTGGGAAGAGAAGTGT	GTGGTTTTGCCCTGGCTGTG
mm_Vcam1	AGTTGGGGATTCGGTTGTTC	CATTCCTTACCACCCCATTG
mm_Cd31	ATCAGCTGCCAGTCCGAA	AGGTCACCTCGAGAGTCTGG
mm_Actb	GAAGCTGTGCTATGTTGCTCTA	GGAGGAAGAGGATGCGGCA
mm_Ppia	ATTTCTTTTGACTTGCGGGC	AGCTAGACTTGAAGGGGAATG
mm_B2m	CCGCCTCACATTGAAATCC	CTCTGCAGGCGTATGTATCAG

Human cells and tissues	Fw	Rev
hs_VIM	ACACACTCAGTGCAGCAATATAT	GGAGTGTCGGTTGTTAAGAACTA
hs_VIM	GCGAGGAGAGCAGGATTTCTC	ACCAGAGGGAGTGAATCCAGA
hs_VEGFA	AAGGAGGAGGGCAGAATCAT	CCAGGCCCTCGTCATTG
hs_KDR (VEGFR2)	ATGACATTTTGATCATGGAGC	CCCAGATGCCGTGCATGAG
hs_CD274 (PD-L1)	CTGAGTGGTAAGACCACCAC	CTGTATGGTTTTCCTCAGGATCTA
hs_CDH5 (VE-cadherin)	ATGATGCCCTCGTTGTGG	CCAGACCAAGTACACATTTGTC
hs_CDH5 (VE-cadherin)	TCCCGGAGCAGAAGACGTC	GAGAAAAGAAAGAGAGCATGGATTG
hs_ICAM1	GGCCGGCCAGCTTATACAC	TAGACACTTGAGCTCGGGCA
hs_VCAM1	TCAGATTGGAGACTCAGTCATGT	ACTCCTCACCTTCCCGCTC
hs_VCAM1	TAACGGGGAGCTACAGCC	CAGCCTGGTTAATTCCTTCAC
hs_ACTB	CATTCCAAATATGAGATGCATT	CCTGTGTGGACTTGGGAGAG
hs_PPIA	CTCGAATAAGTTTGACTTGTGTTT	CTAGGCATGGGAGGGAACA
hs_B2M	TCCATCCGACATTGAAGTTG	CGGCAGGCATACTCATCTT

Supplementary Table 8 - Data sets

**Figure 1C**	Cancer type	Species	Reference	
GSE89287	Colorectal cancer	Human	Zuurbier	https://www.ncbi.nlm.nih.gov/geo/query/acc.cgi?acc=GSE89287
	Glioma	Human	Pen	https://pubmed.ncbi.nlm.nih.gov/17266141/
GSE90459	Glioma	Mouse	Kim	https://www.ncbi.nlm.nih.gov/geo/query/acc.cgi?acc=GSE90459
E-MTAB-3949	Glioma	Mouse	Kaoma	https://www.ebi.ac.uk/arrayexpress/experiments/E-MTAB-3949/
	Melanoma	Mouse	In house	Available on request; provided in Source Data file

**Supplementary Figure 1E**				
GSE39528	Colorectal cancer	Marisa	r2.amc.nl	A
	Colorectal cancer	SieberSmith	r2.amc.nl	B
GSE2109	Colorectal cancer	EXPO	r2.amc.nl	C
GSE21458	Colorectal cancer	Dormany	r2.amc.nl	D
	Colorectal cancer	TCGA	r2.amc.nl	E

**Supplementary Figure 6D**			
GSE17538	Colorectal cancer	Smith	https://www.ncbi.nlm.nih.gov/geo/query/acc.cgi?acc=GSE17538
GSE4290	Brain tumors	Sun	https://www.ncbi.nlm.nih.gov/geo/query/acc.cgi?acc=gse4290
GSE65904	Melanoma	Jonsson	https://www.ncbi.nlm.nih.gov/geo/query/acc.cgi?acc=GSE65904
