# Supplementary material for: Extracellular vimentin mimics VEGF and is a target for anti-angiogenic immunotherapy
Source: Nat Commun. 2022 May 23;13:2842. doi: 10.1038/s41467-022-30063-7 (PMC9126915; doi:10.1038/s41467-022-30063-7)
Supplement: Supplementary file 2 — Reporting Summary [file 41467_2022_30063_MOESM2_ESM.pdf]

nature research

Corresponding author(s): JR van Beijnum; AW Griffioen

Last updated by author(s): 31/3/2022

Reporting Summary

Nature Research wishes to improve the reproducibility of the work that we publish. This form provides structure for consistency and transparency in reporting. For further information on Nature Research policies, see our Editorial Policies and the Editorial Policy Checklist.

Statistics

For all statistical analyses, confirm that the following items are present in the figure legend, table legend, main text, or Methods section.

n/a	Confirmed	
☐	☒	The exact sample size (*n*) for each experimental group/condition, given as a discrete number and unit of measurement
☐	☒	A statement on whether measurements were taken from distinct samples or whether the same sample was measured repeatedly
☐	☒	The statistical test(s) used AND whether they are one- or two-sided<br>*Only common tests should be described solely by name; describe more complex techniques in the Methods section.*
☒	☐	A description of all covariates tested
☐	☒	A description of any assumptions or corrections, such as tests of normality and adjustment for multiple comparisons
☐	☒	A full description of the statistical parameters including central tendency (e.g. means) or other basic estimates (e.g. regression coefficient) AND variation (e.g. standard deviation) or associated estimates of uncertainty (e.g. confidence intervals)
☐	☒	For null hypothesis testing, the test statistic (e.g. *F*, *t*, *r*) with confidence intervals, effect sizes, degrees of freedom and *P* value noted<br>*Give P values as exact values whenever suitable.*
☒	☐	For Bayesian analysis, information on the choice of priors and Markov chain Monte Carlo settings
☒	☐	For hierarchical and complex designs, identification of the appropriate level for tests and full reporting of outcomes
☒	☐	Estimates of effect sizes (e.g. Cohen's *d*, Pearson's *r*), indicating how they were calculated

Our web collection on statistics for biologists contains articles on many of the points above.

Software and code

Policy information about availability of computer code

Data collection

Flowcytometry data were collected on a FACSCalibur and analysed with CellQuest Pro software (BD Biosciences), or using the Legendplex Data Analysis Software Suite (Bio-Legend) and on an LSR Fortessa and analysed with FlowJo v10 (BD Biosciences). For tSNE analysis, the Barnes-Hut algorithm was applied under default settings (1000 iterations, perplexity 30).
qPCR data were collected using a CFX96 thermal cycler using CFX Manager Software v3.1 (Bio-Rad)
Surface Plasmon Resonance (SPR) biosensor data were obtained using using Biacore T200 (GE Healthcare).
ELISA data were obtained using a Biotek Synergy HT microplate reader equipped with Gen5 software (Biotek)
Microscopy image acquisition was performed using Leica Application Suite v4.13.10 (Leica), ImageFocus4 (Euromex) or UniversalGrab 6.3 (DCI Labs)

Data analysis

The following software was used for primary data analysis: MS Excel 2010, Rstudio 2021.09.01, build 372, R version 4.1.2. Packages used in R studio were DEseq2 (https://bioconductor.org/packages/release/bioc/html/DESeq2.html) and mMCP counter (https://github.com/cit-bioinfo/mMCP-counter). Statistical analysis was performed with GraphPad Prism 9.3.1.
Overrepresentation analysis for functions and pathways was performed using Webgestalt (http://webgestalt.org/). Gene expression correlation analysis was performed with R2 (https://hgserver1.amc.nl/cgi-bin/r2/main.cgi). Gene set enrichment analysis (GSEA) was performed with GSEA 4.1.0 (https://www.gsea-msigdb.org/gsea/index.jsp) for hallmarks gene sets (h.all.v7.5.1.symbols.gmt). Protein-protein interaction analysis was performed using STRING (https://string-db.org/) and functional enrichment using Enrichr (https://maayanlab.cloud/Enrichr/). Protein-protein interactions were visualised using Cytoscape v3.7.2. Public gene expression data sets were retrieved from NCBI GEO database (https://www.ncbi.nlm.nih.gov/gds/). Protein localization analysis was performed with secretomeP (http://cbs.dtu.dk/services/SecretomeP/) and proteinside (https://www.proteinside.org/).
Digital flow cytometry was performed using Cibersort (https://cibersort.stanford.edu/).
Image J 1.50i was used for specified image analysis using the plugins Angiogenesis Analyzer (http://image.bio.methods.free.fr/ImageJ/?Angiogenesis-Analyzer-for-ImageJ&lang=en) and Colour deconvolution (https://imagej.net/plugins/colour-deconvolution).

Drawings were generated in Adobe Illustrator CS6 or BioRender under agreement number VZ23O8HZ31

For manuscripts utilizing custom algorithms or software that are central to the research but not yet described in published literature, software must be made available to editors and reviewers. We strongly encourage code deposition in a community repository (e.g. GitHub). See the Nature Research guidelines for submitting code & software for further information.

Data

Policy information about availability of data

All manuscripts must include a data availability statement. This statement should provide the following information, where applicable:

- Accession codes, unique identifiers, or web links for publicly available datasets
- A list of figures that have associated raw data
- A description of any restrictions on data availability

Data deposition:
GSE172388 https://www.ncbi.nlm.nih.gov/geo/query/acc.cgi?acc=GSE172388
PXD024426 https://www.ebi.ac.uk/pride/archive/projects/PXD024426

Public data resources used:
GSE89287 https://www.ncbi.nlm.nih.gov/geo/query/acc.cgi?acc=GSE89287
GSE90459 https://www.ncbi.nlm.nih.gov/geo/query/acc.cgi?acc=GSE90459
E-MTAB-3949 https://www.ebi.ac.uk/arrayexpress/experiments/E-MTAB-3949/
GSE17538 https://www.ncbi.nlm.nih.gov/geo/query/acc.cgi?acc=GSE17538
GSE4290 https://www.ncbi.nlm.nih.gov/geo/query/acc.cgi?acc=gse4290
GSE65904 https://www.ncbi.nlm.nih.gov/geo/query/acc.cgi?acc=GSE65904

Nucleotide sequences:
Murine vimentin (NM_011701; https://www.ncbi.nlm.nih.gov/nuccore/NM_011701/)
Dog vimentin (NM_001287023.1; https://www.ncbi.nlm.nih.gov/nuccore/NM_001287023.1)

Field-specific reporting

Please select the one below that is the best fit for your research. If you are not sure, read the appropriate sections before making your selection.

☒ Life sciences ☐ Behavioural & social sciences ☐ Ecological, evolutionary & environmental sciences

For a reference copy of the document with all sections, see nature.com/documents/nr-reporting-summary-flat.pdf

Life sciences study design

All studies must disclose on these points even when the disclosure is negative.

Sample size

For mouse experiments, the following analysis was initially performed:
If we assume (resulting from previous experiments) that treatment (vaccination) is 70% effective and the outcome is tumor growth then the calculation is as follows:
π (treatment group)=0.70, π (control group)=0, k=10.5 at 90% power and α=0.05,
d=0.7, πmean= (0+0.7)/2=0.35
The following formula is filled in: n~(2 x πmean x (1-πmean) x k)/d^2 ~ 9.75
Initial experiments demonstrated already sufficient power with 5 mice per group.

For in vitro experiments, no prior statistical analysis was performed for sample size. Based on experience with these assays, 3 to 5 independent experiments, performed in duplicate or triplicate were are sufficient. For qPCR, in vitro assays were performed on 3 to 5 independent passages (HMEC-1) or donors (HUVEC), and analysed 3 times in independent experiments. Only samples showing appropriate melting curves and relevant Ct values were included in subsequent analysis.

Data exclusions

Experiments were included based on the accurate (pre-established) outcomes of positive and negative controls. Data were excluded if controls were not accurate. In qPCR, if melting curves were not appropriate or if pre-defined Ct values were exceeded, the sample was excluded from analysis.
In addition, when possible outliers were present, a Grubbs test (https://www.graphpad.com/quickcalcs/grubbs1/) was performed to decide about exclusion.

Replication

In vitro experiments were performed in at least 3 to 5 independent experiments, and were replicable. All data of the experiments were included in this report, generally presented as means +/- SEM, with individual data shown.
In vivo mouse experiments were performed once for this study. During the execution of the work presented in this manuscript, we observed excellent reproducibility of vimentin vaccination, used as control vaccine in ongoing studies.
Proteomics, RNAseq, multipanel flowcytometry and surface plasmon resonance analysis were performed once.

Randomization

In vivo experiments: Following tumor inoculation, mice and eggs were randomized according to tumor burden for the passive immunization experiments. For vaccination experiments, mice were randomly assigned to receive control or test vaccine.

	In vitro experiments: No randomization could be applied. In cellular assays, cells from a single passage received different treatments as indicated, and data were normalized to untreated controls to correct for donor variations.
Blinding	For proper execution of the experiments, the investigator required knowledge of the treatment groups, therefore, no blinding was applied

Reporting for specific materials, systems and methods

We require information from authors about some types of materials, experimental systems and methods used in many studies. Here, indicate whether each material, system or method listed is relevant to your study. If you are not sure if a list item applies to your research, read the appropriate section before selecting a response.

Materials & experimental systems

n/a	Involved in the study	
☐	☒	Antibodies
☐	☒	Eukaryotic cell lines
☒	☐	Palaeontology and archaeology
☐	☒	Animals and other organisms
☒	☐	Human research participants
☒	☐	Clinical data
☒	☐	Dual use research of concern

Methods

n/a	Involved in the study	
☒	☐	ChIP-seq
☐	☒	Flow cytometry
☒	☐	MRI-based neuroimaging

Antibodies

Antibodies used	Antibodies used in in vitro and in vivo assays, and for detection of proteins by immunofluorescence, immunoblotting or single-color flowcytometry and ELISA are detailed in Supplementary Table 4. Antibodies used in immunohistochemical stainings, along with protocol details, are presented in Supplementary Table 5. Antibody panels used for immunoprofiling by flow cytometry are detailed in Supplementary Table 6.
Validation	Antibodies were chosen based on published work, or on information provided by the supplier for suitability. Guidance of the supplier and published work was followed for in-house optimization of usage of antibodies. For imaging (immunohistochemistry, immunofluorescence), multiple dilutions of primary antibodies were tested, ranging from 1:50 to 1:500, to determine the optimal dilution for highest signal to background. For immunohistochemistry, different antigen retrieval methods were tested. For ELISA, different anti-vimentin antibodies (V9, RV202 and E-5) were tested in pilot assays on recombinant vimentin protein to determine optimal dilution. Antibody panels for flow cytometry were designed in close collaboration with the supplier, Bio-Legend. TE6 (in-house produced monoclonal antibody) and vimentin nanobody (QVQ) were additionally validated by ELISA on recombinant vimentin.

Eukaryotic cell lines

Policy information about cell lines

Cell line source(s)	RF24 ((immortalized human vascular endothelial cells) – Gift, available in-house. https://pubmed.ncbi.nlm.nih.gov/7813621/<br>HMEC-1 (immortalized human vascular endothelial cells) - ATCC CRL-3243<br>Jurkat (immortalized human T-lymphocytes) - ATCC TIB-152<br>786-O (human renal cell carcinoma) - ATCC CRL-1932<br>MDA-MB-231 (human breast carcinoma) - ATCC CRM-HTB-26<br>A2780 (human ovarian carcinoma) - ECACC 93112519<br>HCT116 (human colorectal carcinoma) - ATCC CCL-247<br>B16F10 (mouse melanoma) - ATCC CRL-6475<br>SVEC (mouse endothelial cells) - ATCC CRL-2181<br>CT26 (mouse colorectal carcinoma) - ATCC CRL-2638<br><br>Cells were originally obtained from ATCC, ECACC or available in-house as stated above
Authentication	No additional authentication was performed.
Mycoplasma contamination	Cells were routinely tested for mycoplasm and discarded when positive.
Commonly misidentified lines<br>(See ICLAC register)	None

Animals and other organisms

Policy information about studies involving animals; ARRIVE guidelines recommended for reporting animal research

Laboratory animals	8-week old female C57BL/6J (C57BL/6OlaHsd) mice or BALB/c mice (BALB/cOlaHsd) (Envigo, Horst, The Netherlands) were used in the experiments. Mice were allowed to acclimatize 2 weeks, housed at ambient temperature (20-24oC) and humidity (45 to 65%),

	with a 12/12h light dark cycle and were fed ad libitum.<br>Dogs included in the study are domestic dogs that enrolled voluntarily by owner consent, and are not considered laboratory animals. Dog patients with spontaneous (recurrent) transitional cell carcinoma (TCC) of the bladder were recruited within their own veterinary practice. Upon owner consent, dogs were included in the study to receive vimentin vaccinations and followed regular monitoring schedules within their own veterinary practice. Dogs followed normal domestic routine with their owners during the study. Patient details are described in Supplementary Table 2 and summarized in Table 1.<br>For required reporting completeness, the study population consisted of 10 dog patients, included between February 20th 2020 and February 20th 2021, and which had received at least three vaccinations before June 1st 2021. Recurrent TCC patients included: Galgo español (11.9y, female spayed), Podenco ibicenco (13.9y, male castrated), German hunting terrier (11.1y, male castrated), Yorkshire terrier (10.3y, female spayed). Primary TCC patients included: Bernese mountain dog (7.0y, male castrated), Boomer (9.9y, female spayed), Small dutch waterfowl dog (8.9y, female intact), Lagotto romagnolo (14.2y, female spayed), Labrador retriever (11.8y, female spayed), Australian shepherd (7.2y, male castrated).
Wild animals	No wild animals were used in this study
Field-collected samples	No field collected samples were used in this study
Ethics oversight	Studies were approved by the local Animal Ethics Committee of the VU University and the national Central Animal Experiments Committee (CCD); AngL13-02, AngL14-01, AVD114002016576, AVD1140020173104, AVD11400202011305.

Note that full information on the approval of the study protocol must also be provided in the manuscript.

Flow Cytometry

Plots

Confirm that:

☒ The axis labels state the marker and fluorochrome used (e.g. CD4-FITC).

☒ The axis scales are clearly visible. Include numbers along axes only for bottom left plot of group (a 'group' is an analysis of identical markers).

☒ All plots are contour plots with outliers or pseudocolor plots.

☒ A numerical value for number of cells or percentage (with statistics) is provided.

Methodology

Sample preparation	Cultured cells were trypsinized, fixated with 1% PFA. Stained with single antibody and subsequently with FITC or A488 conjugated secondary antibody, as detailed in the supplemental tables. Single-cell suspensions from freshly excised tumors is described in the supplemental materials and methods, and antibody details are presented in Supplementary Tables 4 and 6
Instrument	FACS Calibur (BD), LSR Fortessa (BD)
Software	CellQuestPro, FlowJo v10
Cell population abundance	Flowcytometry was performed to measure protein level in cultured homogeneous cell populations, before and after treatment as indicated. Profiling of immune cell subsets was performed on single cell suspensions created from B16F10 tumors grown in mice
Gating strategy	Cell debris was gated out on FSC/SSC and FL-1 signal (MFI) was adjusted in such way that conjugate control MFI was around 5. See figure S1F. Gating for profiling of immune cell subsets in tumors is detailed in the Supplementary Figures 8 and 9.

☒ Tick this box to confirm that a figure exemplifying the gating strategy is provided in the Supplementary Information.
